# Missing Value Imputation With Low-Rank Matrix Completion in Single-Cell RNA-Seq Data by Considering Cell Heterogeneity

**DOI:** 10.3389/fgene.2022.952649

**Published:** 2022-07-14

**Authors:** Meng Huang, Xiucai Ye, Hongmin Li, Tetsuya Sakurai

**Affiliations:** ^1^ Department of Computer Science, University of Tsukuba, Tsukuba, Japan; ^2^ Center for Artificial Intelligence Research, University of Tsukuba, Tsukuba, Japan

**Keywords:** single-cell RNA-seq, dropout, imputation, low-rank matrix completion, precision medicine

## Abstract

Single-cell RNA-sequencing (scRNA-seq) technologies enable the measurements of gene expressions in individual cells, which is helpful for exploring cancer heterogeneity and precision medicine. However, various technical noises lead to false zero values (missing gene expression values) in scRNA-seq data, termed as dropout events. These zero values complicate the analysis of cell patterns, which affects the high-precision analysis of intra-tumor heterogeneity. Recovering missing gene expression values is still a major obstacle in the scRNA-seq data analysis. In this study, taking the cell heterogeneity into consideration, we develop a novel method, called single cell Gauss–Newton Gene expression Imputation (scGNGI), to impute the scRNA-seq expression matrices by using a low-rank matrix completion. The obtained experimental results on the simulated datasets and real scRNA-seq datasets show that scGNGI can more effectively impute the missing values for scRNA-seq gene expression and improve the down-stream analysis compared to other state-of-the-art methods. Moreover, we show that the proposed method can better preserve gene expression variability among cells. Overall, this study helps explore the complex biological system and precision medicine in scRNA-seq data.

## Introduction

Single-cell RNA-sequencing (scRNA-seq) technologies have revolutionized the throughput and resolution of bulk RNA sequencing in transcriptome studies (Tal 2014; Jaitin et al., 2014; Gierahn et al., 2017; Zheng GXY. et al., 2017). The scRNA-seq can characterize the gene expression of individual cells without ignoring the potential cell heterogeneity (Macosko et al., 2015). In recent years, the advancements of scRNA-seq have significantly enhanced the classification of cell subtypes (Usoskin et al., 2015; Zeisel et al., 2015), the quantification of gene expressions (Xue et al., 2013; Treutlein et al., 2014), and the identification of differentially expressed genes (Lee et al., 2014; Kim et al., 2015). The scRNA-seq analysis is also used in other studies, such as the immune system (Bjorklund et al., 2016; Papalexi and Satija, 2018), the brain neuronal mechanisms (Zeisel et al., 2015; Lake et al., 2016; Lake et al., 2018), and the cancer-related diseases (Zheng C. et al., 2017: Guo et al., 2018; Peng et al., 2019; Zhang et al., 2020). As the precision medical technology continues to develop, more researchers are using the scRNA-seq data analysis to explore cancer heterogeneity. However, the sparse gene expression matrix limits the performance of scRNA-seq technology to provide accurate measurements in single cells. For example, the zero counts of the typical matrix may have exceeded 90% of the counts in the droplet-based datasets (Huang et al., 2018; Van-Dijk et al., 2018). Most zero counts are produced by the partially low expression of genes, the low-sequencing depth of cells, and dropout events (Kharchenko et al., 2014; Lun et al., 2016; Ziegenhain et al., 2017; Patruno et al., 2021). Especially, the dropout events may lead to non-biological zero counts (missing gene expression values), which hinder a high-precision analysis in scRNA-seq data (Gong et al., 2018; Huang et al., 2018; Li and Li, 2018; Van-Dijk et al., 2018).

Recently, several imputation methods have been proposed to address the problems of missing gene expression values in scRNA-seq data. We can roughly divide these methods into four categories: model-based, smoothing-based, deep learning-based, and matrix theory-based methods. For example, Li et al. proposed scImpute (model-based) to automatically identify dropouts and detect outlier cells with additional information about the cell types (Li and Li, 2018). Huang et al. developed SAVER (model-based) by utilizing a Markov chain Monte Carlo algorithm to infer all the parameters, but results in the extremely high computational complexity (Huang et al., 2018). Van-Dijk et al. put forward MAGIC (smoothing-based) to impute missing gene expression values by projecting the data into a low-dimensional space (Van-Dijk et al., 2018). Gong et al. proposed DrImpute (smoothing-based) by using the average values of the gene expression in similar cells (Gong et al., 2018). However, the smoothing-based methods reduce the gene expression variability between cells. Taking advantages of the superior performance of the neural network, Arisdakessian et al. developed DeepImpute (deep learning-based) to impute missing gene expression values by learning the scRNA-seq data patterns (Arisdakessian et al., 2019), which leads to the unexplainable problem for the scRNA-seq data analysis. Linderman et al. put forward ALRA (matrix theory-based) to impute the missing values for the expressed genes (non-zero values) by using matrix approximation, which preserves the biological meaning of non-expressed genes (Linderman et al., 2018). Although these methods can impute missing gene expression values at a certain level, they have not considered the cell heterogeneity. It is still a challenge to recover missing gene expression values more effectively in scRNA-seq data. Previous studies (Narayanamurthy et al., 2019; Nguyen et al., 2019; Kummerle and Verdun, 2021; Zilber and Nadler, 2021) have shown that the low-rank matrix can recover missing values based on a few observable entries due to its low-rank structure. Considering this, we apply low-rank matrix completion to missing value imputation in scRNA-seq data.

This study proposes a novel scRNA-seq imputation method, called single cell Gauss–Newton Gene expression Imputation (scGNGI), to impute the missing gene expression values in scRNA-seq data. It associates the cell heterogeneity with the low-rank matrix, and regards dropout events as the main source of missing values (Lun et al., 2016; Ziegenhain et al., 2017). Gauss–Newton linearization is applied to the approximation iteration of sparse gene expression matrices in the proposed scGNGI method. We conduct a large number of experiments on the real and simulated scRNA-seq datasets by comparing with six state-of-the-art methods. The obtained experiment results show that our method, scGNGI, is an effective tool to recover the biologically meaningful expression of genes in scRNA-seq data, improve the low-dimensional representation and clustering analysis, and recover the gene-wise relationship. We also evaluate its performance for the imputation of marker gene expression and the preservation of gene expression variability among cells. All in all, this study helps explore complex biological systems, cancer-related diseases, and precision medicine in scRNA-seq data.

## Materials

The gene expression data analysis helps evaluate the imputation performance in scRNA-seq data. Real human and mice datasets were used for this experiment. Furthermore, we also use simulated scRNA-seq datasets to evaluate the proposed method extensively.

### Real scRNA-seq Datasets

We collected two real scRNA-seq datasets from the studies of human embryonic stem cells (ESCs), and mouse arcuate nucleus and median eminence cells (ANMECs), respectively.

#### Human ESC scRNA-seq Dataset

The first real data set is from a human study, where Chu et al. dissected the human embryonic stem cell entry into endoderm progenitors (Chu et al., 2016). In this human ESC study, four expected count matrices are provided. We only used one of them, which contains 1,018 single cells measured on the lineage-specific progenitor cells. The 1,018 single cells are divided into seven known cell types: undifferentiated H1 and H9 ESCs, definitive endoderm derivative cells (DECs), endothelial cells (ECs), foreskin fibroblasts (HFFs), neuronal progenitor cells (NPCs), and trophoblast-like cells (TBs). To some extent, these different cell types reveal the heterogeneity for each type of progenitors. These gene expression measurements can be regarded as the “Cell Type”. We downloaded the expected count matrix from the data repository NCBI Gene Expression Omnibus (GEO access number: GSE75748). This count matrix is analyzed as one table with columns representing the cells and rows representing the genes. We obtained 1,018 samples (single cells) and 19,097 attributes (genes).

#### Mouse ANMECs scRNA-seq Dataset

The second real dataset is from a mouse study about arcuate hypothalamus and median eminence cell types, where Chu et al. performed the single-cell RNA-seq for two adult male RIP-Cre mice (Saunders et al., 2017). The gene counts matrix contains 25 cells without the detailed subpopulation information. These gene expression measurements can be viewed as the “RIP-Cre”. We obtained the expected count matrix from the data repository NCBI Gene Expression Omnibus (GEO access number: GSE90806). We obtained 25 samples (single cells) and 30,927 attributes (genes).

### Simulated scRNA-Seq Datasets

We used the R package Splatter (v1.17.1) (Zappia et al., 2017) to generate three simulated scRNA-seq datasets with three different dropout rates (56.3%, 50.2%, and 13.4%). The R function splatSimulate was used by setting the different number of genes, cells, and cell types. Consequently, we obtained “Simulated Data 1”, “Simulated Data 2”, and “Simulated Data 3” for scRNA-seq count data, respectively. “Simulated Data 1” contains 18,000 genes and 1,000 cells with five cell types, “Simulated Data 2” includes 13,000 genes and 700 cells with four cell types, and “Simulated Data 3” has 1,000 genes and 800 cells with three cell types. In the R function (splatSimulate) settings, we used the default values to generate the ground truth for the remaining parameters.

### Data Pre-Processing

To reduce the error produced by the technical noise in the scRNA-seq dataset, we performed pre-processing for all the data. Firstly, we removed the duplicate genes for all real and simulated scRNA-seq datasets. Then, we filtered out genes expressed in <5 cells, and cells with expressed genes <200 for all datasets. Next, we performed the log-transformation, log(count + 1) , for all filtered data, which reduces the variances in the raw read counts. Finally, we obtained Cell Type data with 17,191 genes and 1,018 cells, RIP-Cre data with 11,217 genes and 25 cells, Simulated Data 1 with 17,392 genes and 1,000 cells, Simulated Data 2 with 12,543 genes and 700 cells, and Simulated Data 3 with 995 genes and 800 cells. To obtain the artificial missing data, we randomly masked the 2%, 5% and 10% non-zero gene expressions for four datasets: Cell Type, RIP-Cre, Simulated Data 1, and Simulated Data 2. To illustrate the effectiveness of the proposed method on a large number of missing values, the 10 and 35% non-zero gene expressions were randomly masked in Simulated Data 3. Note that we can obtain the corresponding ground truth from the raw data for these artificial missing data to evaluate the performance of imputation methods in the experiments.

## Methods

### Notations

The single-cell gene expression matrix is denoted as $X=\left(x_{ij}\right)\in {\mathbb{R}}^{m\times n}$ , where m is the number of genes, and n is the number of cells. The updating gene expression matrix is denoted as $X^{\prime }=\left(x_{ij}^{\prime }\right)$ in the optimization process, and the final imputed gene expression matrix is denoted as $X^{*}=\left(x_{ij}^{*}\right)$ . The number of cell types and non-zero gene expression values are denoted as c and d , respectively. Furthermore, f refers to the imputation operator, and ε is regarded as the linear measurement.

### Proposed Method

**FIGURE 1:**
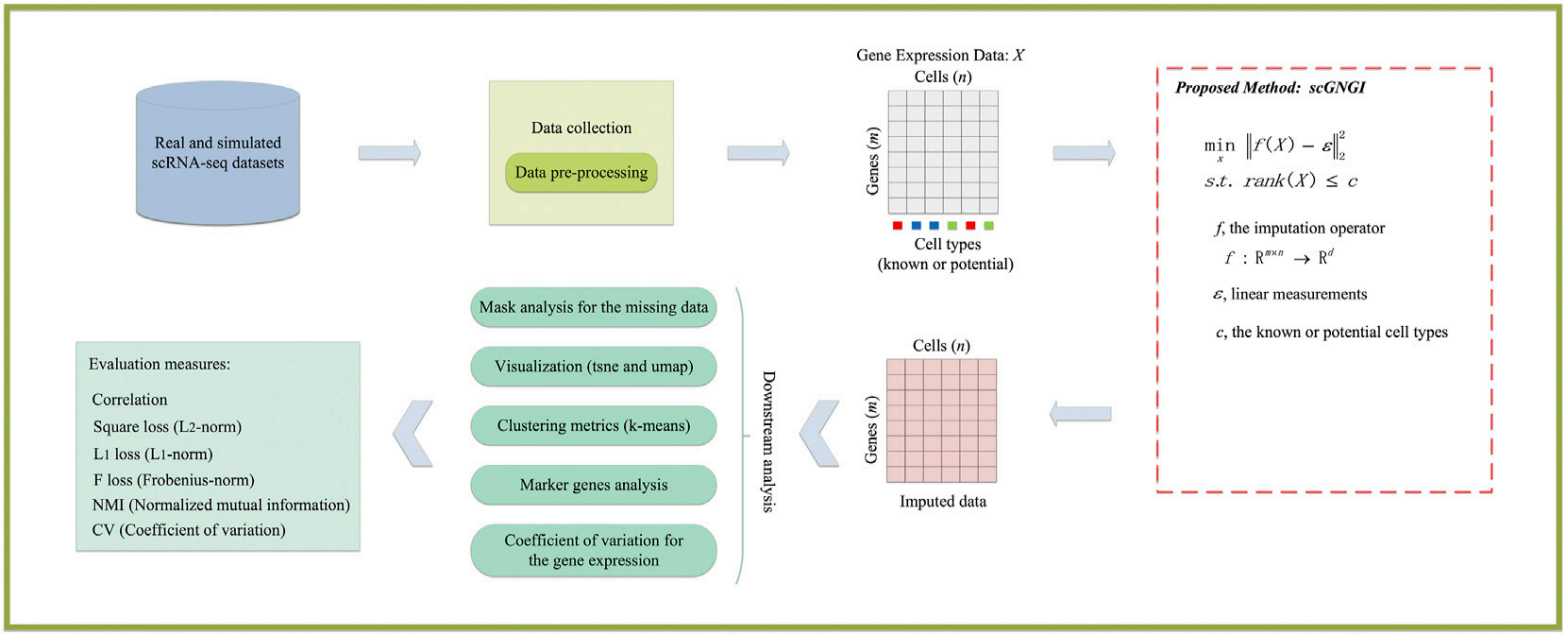
Overview of the proposed scGNGI for single-cell RNA-seq imputations and downstream evaluations.

To impute the missing values of scRNA-seq data, we proposed a novel scRNA-seq imputation method i.e., scGNGI, by using the low-rank matrix completion. The overview of gene expression imputation using the proposed scGNGI method is shown in Figure 1. Firstly, these scRNA-seq datasets are preprocessed to obtain the gene expression data X ; then, X is inputted into the proposed scGNGI, which produces the imputed data. Finally, the imputed gene expression matrix can be used to perform the downstream analysis including the mask analysis for missing data, visualization of cell types, clustering analysis, marker gene analysis, and coefficient of variation for the gene expression.

#### Imputation Operator and Linear Measurements

For the gene expression matrix $X\in {\mathbb{R}}^{m\times n}$ , the set of non-zero gene expression values (observed entry) are denoted as $\Omega =\left\{x_{ij}|x_{ij}\neq 0\right\}$ (|Ω|=d) . Let ID={(i,j)} denotes the index of non-zero values in X . Given the decomposition $X\;\;=\;U\;V^{\text{T}}$ with $U\;\;\in \;{\mathbb{R}}^{m\times r}$ and $V\;\;\in \;{\mathbb{R}}^{n\times r}$ , (m+n)d variables are involved. To recover the missing gene expression values more accurately, we set the imputation operator f and the linear measurement ε. The imputation operator is a linear map $f:\;{\mathbb{R}}^{m\times n}\;\to \;{\mathbb{R}}^{d}$ , which is to extract d non-zero entries from gene expression matrix X . These extracted *d* non-zero gene expression values are compressed into a vector as the output of the imputation operator; for example, $f\left(X\right)\;\;=\;f\left(U\;V^{\text{T}\;}\right)\;\in \;{\mathbb{R}}^{d}$ . Since the d non-zero values are updated after iteration, we use a sampling operator $\sigma_{\Omega }$ to extract the d observed gene expression values corresponding to the position of the *d* non-zero values in X . The output of $\sigma_{\Omega }$ is set as the linear measurement ε . For instance, $\varepsilon =\;\sigma_{\Omega }\left(\tilde{X}\right)\;=\;\left\{e_{ij}\;|\;\left(i,j\right)\in ID\right\}\;\in \;{\mathbb{R}}^{d}$ , where ε is a vector of size d , $\tilde{X}=X+\beta U_{t}V_{t}^{\text{T}}$ is the updated decompositions of X on $U_{t}$ and $V_{t}$ , and β is the hyper-parameter.

#### Mathematical Formulation of scGNGI

The low-rank matrix completion is widely used for recovering lost information (Nguyen et al., 2019; Kummerle and Verdun, 2021; Zilber and Nadler, 2021), where the missing data are usually estimated by using the low-rank structure of the known data for highly sparse matrices. To consider the cell heterogeneity, we associate the number of cell types and the rank of gene expression X together. Here, we designed a single cell Gauss–Newton Gene expression Imputation (scGNGI), which utilizes the low-rank structure and cell heterogeneity to obtain the optimal approximation of missing data in scRNA-seq data.

The proposed scGNGI is to minimize the differences between the imputation operator and the linear measurements, which is formalized as follows:

$$\begin{array}{c} \underset{X}{\min}{\left\|f\left(X\right)-\varepsilon \right\|}_{2}^{2}\\ s.t.\;\;rank\left(X\right)\;\leq c, \end{array}$$ (1)

where f is the imputation operator ( $f:\;{\mathbb{R}}^{m\times n}\;\to \;{\mathbb{R}}^{d}$ ), ε is the linear measurements ( $\varepsilon \;\;\in \;{\mathbb{R}}^{d}$ ), c is the known or potential cell types.

### Optimization Solution

To solve the optimization problem in Eq. 1, we consider the decomposition $X\;=\;U\;V^{T}$ . Equation 1 is equivalent to

$$\underset{\left(U,V\right)}{\min}{\left\|f\left(U\;V^{T\;}\right)-\varepsilon \right\|}_{2}^{2}.$$ (2)

Gauss–Newton linearization is applied to the approximation iteration of the sparse gene expression matrix. We use the Singular Value Decomposition (SVD) algorithm to obtain the initial estimates $\left(U_{1},V_{1}\right)$ . Next, we acquire an update (ΔU,ΔV) , which minimizes Eq. 2. Given $\left(U_{2},V_{2}\right)=\left(U_{1}+\Delta U,\;V_{1}+\Delta V\right)$ , Eq. 2 can be equivalently written as

$$\underset{\left(\Delta U,\;\Delta V\right)}{\min}{\left\|f\left(U_{1}V_{1}^{\text{T}}+U_{1}\Delta V^{\text{T }}\mathrm{+\Delta}UV_{1}^{\text{T}}+\Delta U\Delta V^{\text{T}}\right)-\varepsilon \right\|}_{2}^{2}.$$ (3)

The second order term $\Delta U\Delta V^{\text{T}}$ can be neglected, which yields the general scheme as follows:

$$\begin{array}{c} \left(\text{Δ}U_{1},\text{Δ}V_{1}\right)=\underset{\text{Δ}U,\text{Δ}V}{\arg\min}{\left\|f\left(U_{1}V_{1}^{T}+U_{1}\text{Δ}V^{T}+\text{Δ}UV_{1}^{T}\right)-\varepsilon \right\|}_{2}^{2},\\ \left(U_{2},V_{2}\right)=\left(U_{1}+\text{Δ}U_{1},V_{1}+\text{Δ}V_{1}\right). \end{array}$$ (4)

To make a better optimization, we design a family of solutions of Eq. 4 by using a scalar as the hyper-parameter. By changing the variables to be optimized $\text{Δ}U=U-\frac{1+\beta }{2}U_{1},\text{ Δ}V=V-\frac{1+\beta }{2}V_{1}$ in Eq. 4, we get

$$\begin{array}{c} \left(U_{1}^{\prime },V_{1}^{\prime }\right)=\underset{U,V}{\arg min}{\left\|f\left(U_{1}V^{T}+UV_{1}^{T}-\beta U_{1}V_{1}^{T}\right)-\varepsilon \right\|}_{2}^{2},\\ \left(U_{2},V_{2}\right)=\left(\frac{1-\beta }{2}U_{1}+U_{1}^{\prime },\frac{1-\beta }{2}V_{1}+V_{1}^{\prime }\right). \end{array}$$ (5)

In this work, the procedure of the proposed scGNGI is summarized as in Algorithm 1. The initial $\left(U_{1},V_{1}\right)$ are calculated by the solution of SVD on the gene expression matrix X . Since the LSQR algorithm (Paige and Saunders, 1982) can find the least-squares solution to a large, sparse, and linear system of equations, we use it to solve the optimal solution of the least squares for $E_{t}^{\prime }$ . In general, the LSQR algorithm is implemented in some standard packages. During the optimization process shown in Eq. 5, we tried to explore different optimization solutions utilizing different β values. Empirically, the solutions with β=1 had the better performance.

In the t+1 iteration, we obtained the optimal estimate values $X^{\prime }=\left(x_{i,j}^{\prime }\right)=U_{\text{t}+1}V_{t+1}^{T}.$ Obviously, the value is non-negative in the gene expression matrix. Therefore, we define

$$S=\left(s_{i,j}\right)=\{\begin{array}{cc} 0 & \;\mathrm{if}\;\;x_{i,j}^{\prime }<0\\ x_{i,j}^{\prime } & \;\mathrm{otherwise}, \end{array}$$ (6)

where i=1, 2,…,m and j=1, 2,…,n . Since the observed data of the gene expression matrix X are usually more accurate, we only impute the missing data. Thus, we define

$$X^{\text{∗}}=\left(x_{i,j}^{\text{∗}}\right)=\{\begin{array}{c} s_{i,j}\;\;\mathrm{if}\;s_{i,j}\notin \Omega \\ x_{i,j}\;\;\mathrm{if}\;x_{i,j}\in \Omega , \end{array}$$ (7)

where Ω  is the non-zero gene expression values in the gene expression matrix X . Finally, we obtain the optimal imputed gene expression matrix $X^{\text{*}}$ .

Algorithm 1 The proposed scGNGI method.

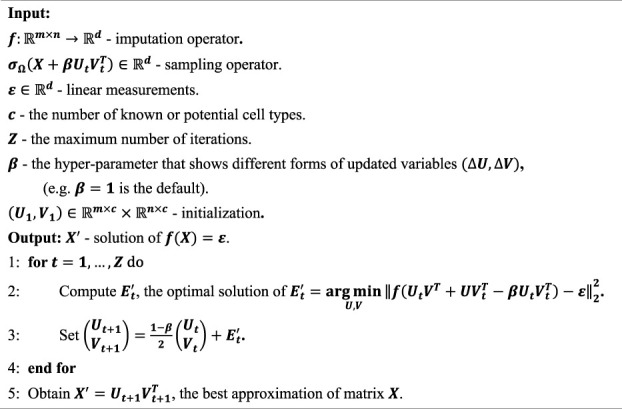

## Experiments and Results

### Experimental Settings

#### Evaluation Metrics

To evaluate the imputation accuracy of the proposed scGNGI method, we quantify the consistency between imputed data and full data by using four metrics, which are Frobenius Error (FE), Correlation (Cor), Mean Squared Error (MSE), and L1 Norm (L1).

FE is defined as follows:

$$FE=\frac{\sqrt{\sum_{i=1}^{m}\sum_{j=1}^{n}{\left|\left(X_{i,j}^{*}-X_{i,j}\right)*M\right|}^{2}}}{\sqrt{\sum_{i=1}^{m}\sum_{j=1}^{n}{\left|X_{i,j}\right|}^{2}}}$$ (8)

where m is the number of genes, n is the number of cells, $X^{\text{*}}$ is the imputed gene expression matrix, X is the gene expression matrix, and the indicator matrix is $M=\left(m_{i,j}\right),\;\;m_{ij}\in \left(0,1\right)$ , that indicates the missing entries in scRNA-seq data.

Furthermore, $X^{\text{*}}\;$ and X are transformed to ${\hat{X}}^{\text{*}}$ and $\hat{X}$ , respectively. ${\hat{X}}^{\text{*}}=\;X^{\text{*}}\text{*}M$ and $\hat{X}=X\text{*}M$ . Here, ${\hat{X}}^{\text{*}}\in {\mathbb{R}}^{m\times n}$ and $\hat{X}\in {\mathbb{R}}^{m\times n}$ , where m is the number of rows with X , and n is the number of columns with X .

Cor is defined as follows:

$$\text{Cor}=\frac{\mathrm{cov}\left({\hat{X}}^{\text{∗}},\hat{X}\right)}{\sigma_{\hat{X}\text{∗}}\sigma_{\hat{X}}}=\frac{E\left[\left({\hat{X}}^{\text{∗}}-\mu_{\hat{X}\text{∗}}\right)\left(\hat{X}-\mu_{\hat{X}}\right)\right]}{\sigma_{\hat{X}\text{∗}}\sigma_{\hat{X}}}\;,$$ (9)

where c ov , σ , and μ are the covariance, standard deviation, and mean values of the samples, respectively.

MSE is defined as follows:

$$MSE=\frac{1}{mn}\sum_{i=1}^{mn}{\left({\hat{X}}^{\text{∗}}-\hat{X}\right)}^{2}$$ (10)

L1 is defined as follows:

$$L1=\frac{1}{mn}\sum_{i=1}^{mn}\left|{\hat{X}}^{*}-\hat{X}\right|$$ (11)

To evaluate the effectiveness of scGNGI imputation for cell clustering, Normalized Mutual Information (NMI) is defined to measure the consistency between estimated and predefined cell clusters in scRNA-seq data. Let $U^{\prime }=\left\{u_{1}^{\prime },u_{2}^{\prime },\ldots ,u_{k}^{\prime }\right\}$ and $V^{\prime }=\left\{v_{1}^{\prime },v_{2}^{\prime },\ldots ,v_{k}^{\prime }\right\}$ denote the estimated and true clustering partition across k class, respectively.

NMI is defined as follows:

$$NMI=\frac{2I\left(U^{\prime },V^{\prime }\right)}{H\left(U^{\prime }\right)+H\left(V^{\prime }\right)}\;.$$ (12)

To measure gene expression variation between cells before and after imputation, Coefficient of Variation (CV) is defined to evaluate different imputation methods.

CV is defined as follows:

$$CV=\left|\frac{\mu_{X_{k_{i}}^{\prime \prime }}}{\sigma_{X_{k_{i}}^{\prime \prime }}}\right|,$$ (13)

where i∈(1,2,…,c) , c is the number of cell types, $k_{i}$ is the number of cells from cell type i , $X_{k_{i}}^{\prime \prime }$ represents the gene expression of cell type i , and μ and σ are the mean values and standard deviation of gene expression, respectively.

#### Parameter Settings

In the proposed scGNGI method, there is an important hyperparameter β in Eq. 5 to control the update of ΔU and ΔV . Empirically, the solution of β=1 resulted in better performance. Thus, we set β equal to 1 as the default value in the experiment. In Eq. 1, c is viewed as the number of known cell types. Empirically, c is set to five for the scRNA-seq data of unknown cell types, where c  = 5 represents the number of potential cell types. All experiments of the proposed scGNGI and other methods were run on the four NVIDIA Tesla Ampere A100-PCIe-40 GB GPUs and Ubuntu18.04 system.

### Mask Analysis for the Missing Data

**FIGURE 2:**
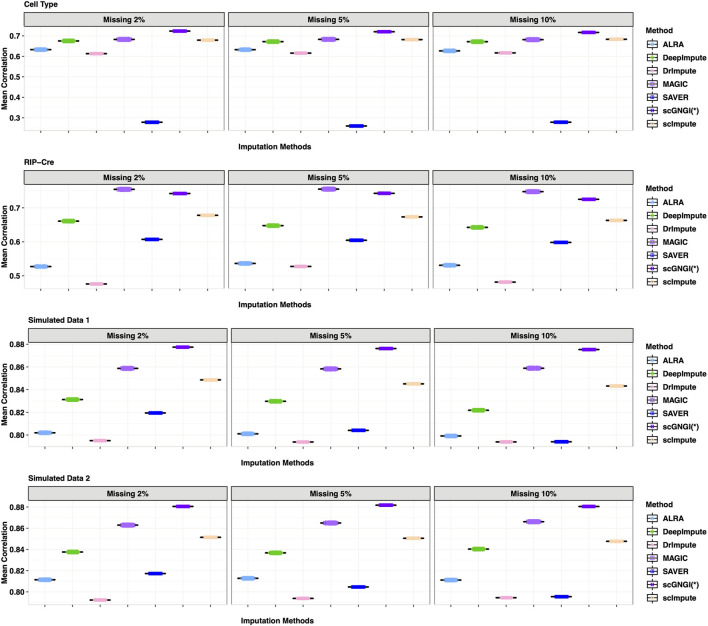
Correlation between the masked truth and imputed values by using different methods in the scRNA-seq data masking experiments. Rows represent the four different datasets (Cell Type, RIP-Cre, Simulated Data 1, and Simulated Data 2). Columns represent three different masking percentages (2%, 5, % and 10%). Boxplots represent the correlation values from 60 masking replicates, where the correlation of each cell is calculated in turn and the mean correlation values are plotted across cells.

To assess the imputation accuracy, we randomly mask non-zero gene expression values through data masking experiments to compare the recovering performance of different methods. For four scRNA-seq datasets (Cell Type, RIP-Cre, Simulated Data 1, and Simulated Data 2), we randomly obtained the non-zero gene expression of the observed data with masking percentages 2%, 5%, and 10%, respectively. These gene expression values were masked to be zero values, which can generate a masked matrix. For the newly generated gene expression matrix, we applied different imputations to recover the missing values. Subsequently, we computed Cor, FE, MSE, and L1 between estimated and masked values to measure the imputation performance. As shown in Figure 2, we show the results of 60 masking replicates to measure the correlation between imputed and masked values. Generally, the proposed scGNGI outperforms other existing methods in Cell Type, Simulated Data 1, and Simulated Data 2. The scImpute, MAGIC, and DeepImpute follow the performance of the proposed scGNGI closely, while ALRA, DrImpute, and SAVER do not show a wonderful performance. For instance, in Cell Type data, the correlation of the masked truth and estimated values by scGNGI is 0.72 with the masking percentage of 2%, while the results of other methods are 0.67 (scImpute), 0.68 (MAGIC), 0.67 (DeepImpute), 0.63 (ALRA), 0.61 (DrImpute), and 0.27 (SAVER), respectively. In addition, these methods produce similar results for four scRNA-seq datasets with the masking percentage of 2, 5, and 10%. As expected, the performances of the proposed scGNGI and other methods descend slowly as the masking percentage increases. Especially, for the dataset of unknown cell types (RIP-Cre), our method still has a better performance than most methods in Figure 2, even though MAGIC is slightly superior to scGNGI. This is because our method considers the cell heterogeneity and utilizes the number of known cell types as a constraint for the optimization solution. Therefore, this shows the proposed scGNGI method has a better performance, especially for the scRNA-seq data of known cell types.

**FIGURE 3:**
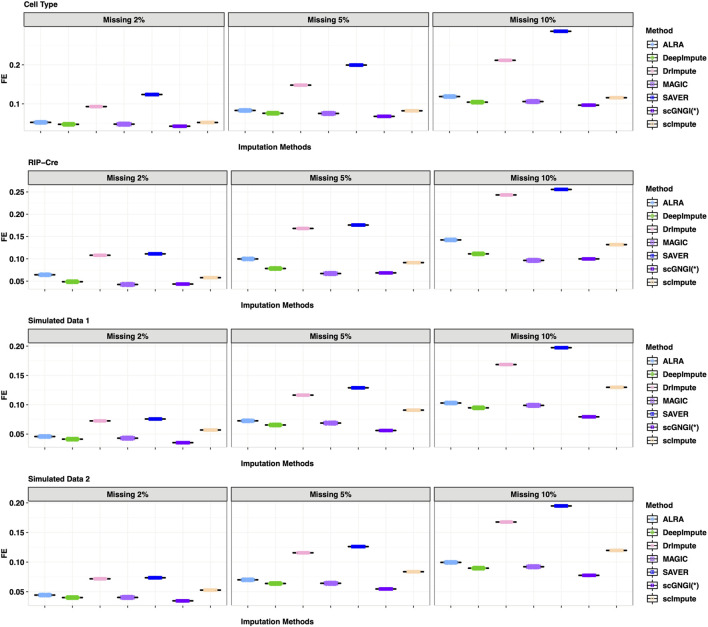
Frobenius error between the masked truth and imputed values by different methods in the scRNA-seq data masking experiments. Rows represent the four different datasets (Cell Type, RIP-Cre, Simulated Data 1, and Simulated Data 2). Columns represent three different masking percentages (2%, 5%, and 10%). Boxplots represent the Frobenius error values from 60 masking replicates, where the Frobenius error of each cell is calculated in turn and the mean Frobenius error values are plotted across cells.

As shown in Figure 3, the results of 60 masking replicates show the Frobenius error between the masked truth and imputed values. Our method obtained the smallest Frobenius error value than all other methods on the four datasets: Cell Type, RIPCre, Simulated Data 1, and Simulated Data 2. For example, in Cell Type data, the Frobenius error value of the masked truth and the estimated values by scGNGI is 0.09 with the masking percentage of 10%, while the results of the other methods are 0.11 (scImpute), 0.10 (MAGIC), 0.10 (DeepImpute), 0.11 (ALRA), 0.21 (DrImpute), and 0.28 (SAVER), respectively. In addition, the MSE and L1 results also show a better performance in our method, as shown in Supplementary Figures S1, S2. These masking experiments show that the proposed scGNGI method can accurately recover the true gene expression for missing values in real and simulated scRNA-seq data.

### Visualization of Different Cell Types

The scRNA-seq data consist of many cell types and the high dropout rate results in the vague differences among cell types. Imputation can help recover cell types for downstream clustering analyses by improving the scRNA-seq data quality. To measure the imputation performance on separating known cell types more accurately, we visualized the imputed data performed by different methods on synthetic datasets (Simulated Data 1, Simulated Data 2, and Simulated Data 3) with different masking percentages (10% and 35%). The t-distributed Stochastic Neighbor Embedding (t-SNE) algorithm (Van-der-Maaten and Hinton, 2008) is applied to visualize the imputed scRNA-seq data with known cell-type labels. To compare the visualization results more reasonably, we also used the Uniform Manifold Approximation and Projection (UMAP) (McInnes et al., 2018) algorithm for visualization.

**FIGURE 4:**
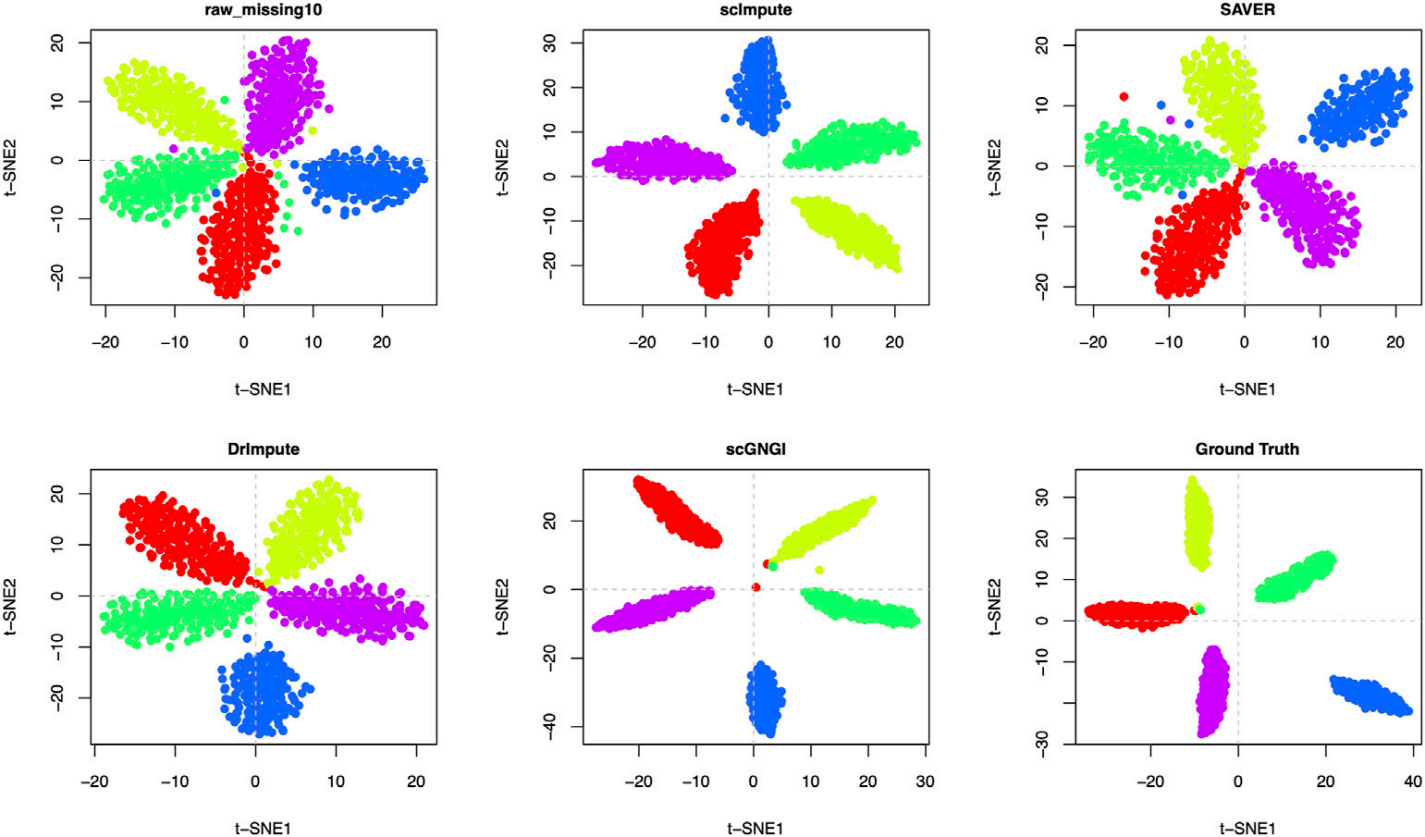
Imputation performance on the Simulated Data 1 with 5 known cell types. Visualization of the cells by the first two t-SNE components on the raw data, missing data, and imputed ones by different methods. Each dot is a single cell, and different colors represent different cell types.

As shown in Figure 4 and Supplementary Figure S3, the proposed scGNGI is superior to other imputation methods on Simulated Data 1 with 5 known cell types. Compared to the visualization of raw data with a 10% masking percentage, SAVER and DrImpute have no obvious improvement, and scImpute slightly improved the scRNA-seq data. However, scGNGI can maintain a similar cell sub-population structure to Ground Truth according to the cell clustering results (Figure 4 and Supplementary Figure S3), which helps separate known cell types. For Simulated Data 2 with 4 known cell types, we can find that scGNGI still outperforms other imputation methods, as shown in Supplementary Figures S4, S5, which is similar to the result of Simulated Data 1.

**FIGURE 5:**
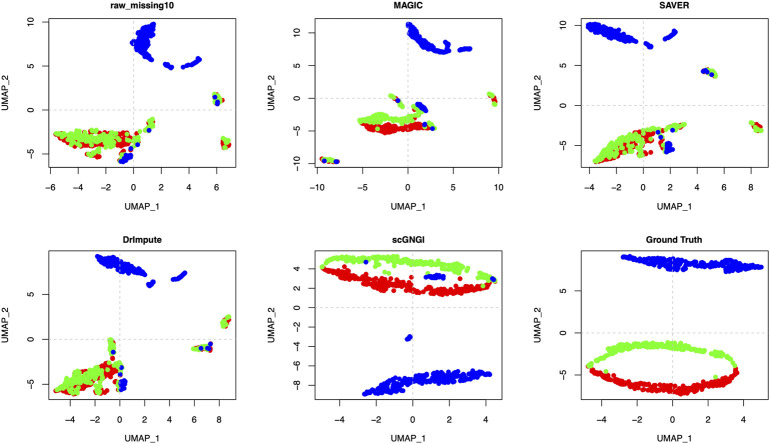
Imputation performance on the Simulated Data 3 with 3 known cell types. Visualization of the cells by the first two UMAP components on the raw data, missing data, and imputed ones by different methods. Each dot is a single cell, and different colors represent different cell types.

To further evaluate the cell sub-population separability, we utilized Simulated Data 3 with 3 known cell types. Lots of zero values cause mixed cell sub-populations in the raw data. The different masking percentages (10% and 35%) make it more difficult to distinguish cell sub-population according to the first visualization plot in Figure 5 and Supplementary Figures S6–S8. However, the imputed Simulated Data three by the proposed scGNGI can help separate the cell cluster. As shown in Figure 5 and Supplementary Figure S6, scGNGI resembles the most to that of Ground Truth compared to other methods. The visualization of the scRNA-seq data imputed by MAGIC, SAVER, and DrImpute shows that many cells from different cell types overlap with each other as raw data with a 10% masking percentage. As can be seen from these visualization results, the cells are divided into different small groups in the same cluster, which does not reflect the cell types. This is inconsistent with the visualization of the cells with similar data structures from the same cell type.

As shown in Figure 5 and Supplementary Figure S6, the visualization of the data imputed by scGNGI suggests that the clear data structures are similar to Ground Truth. For Simulated Data 3 with a 35% masking percentage, our method still outperforms scImpute, SAVER, MAGIC, ALRA, and DrImpute, as shown in Supplementary Figures S7, S8. In Figure 5 and Supplementary Figure S8, we can find that the imputation performance of different methods descends with the increase of masking percentages in raw data. This shows that more missing data may hinder the accurate imputation of scRNA-seq data. However, our methods can still improve the scRNA-seq data, which helps separate many cell types more clearly.

Accordingly, the visualizations by t-SNE and UMAP show that our method can provide higher-quality data by imputing missing values of scRNA-seq data, which makes various cell subpopulations more separable. Compared to other methods, the proposed scGNGI method is more helpful for recovering missing values caused by dropouts and true cell clusters.

### Clustering Analysis of Different Imputation Methods

**FIGURE 6:**
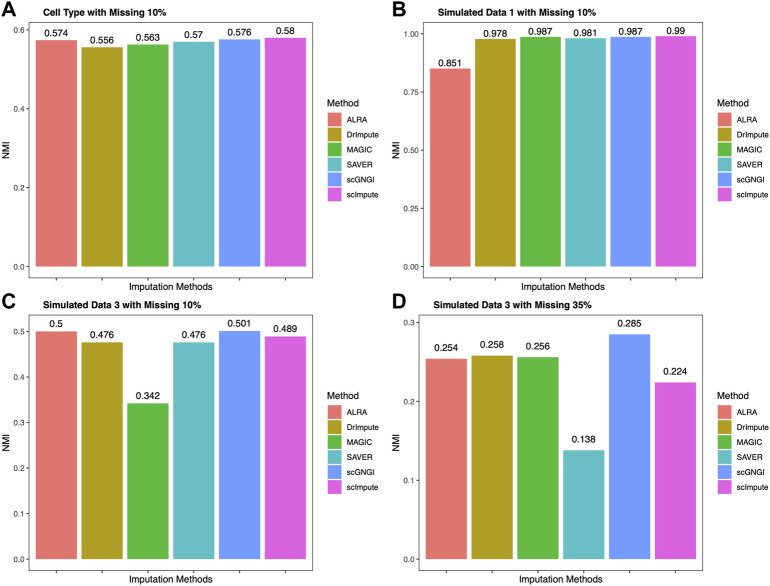
Clustering performance on four scRNA-seq datasets. The results of K-means clustering are measured by the Normalized Mutual Information (NMI) on real and simulated data using different imputation methods.

After obtaining the imputed data, K-means is applied to the clustering cells. Normalized Mutual Information (NMI) is used as the clustering metrics to measure the results on real and simulated datasets. The clustering accuracy is showed in Figure 6. We compared scGNGI and other imputation methods in Cell Type, Simulated Data 1, and Simulated Data 3. As shown in Figure 6A, the NMI obtained by the scGNGI is 0.576 for Cell Type data. Compared with ALRA (0.574), DrImpute (0.556), MAGIC (0.563), and SAVER (0.57), the scGNGI exhibits higher accuracy. For Simulated Data 1, the scGNGI (0.987) outperforms ALRA (0.851), DrImpute (0.978), and SAVER (0.981), and obtains the same clustering accuracy with MAGIC (0.987) in Figure 6B. In addition, one can find that the proposed scGNGI is slightly lower than scImpute according to the clustering accuracy in Figures 6A,B. This shows that scGNGI has better imputation performances in Cell Type and Simulated Data 1 datasets, which helps improve the identification of the cell types.

In Simulated Data 3, we obtained two different scRNA-seq datasets at the missing value ratios of 10 and 35% to evaluate the clustering accuracy of imputed data by different imputation methods. As shown in Figure 6C, the proposed scGNGI reaches the highest accuracy (0.501) compared with ALRA (0.5), DrImpute (0.476), MAGIC (0.342), SAVER (0.476), and scImpute (0.489). Furthermore, our method still maintains the best imputation performance to help identify cell types in Figure 6D. Specifically, the clustering performance of the imputed data by all imputation methods gradually decreases as the missing value ratio increases, as shown in Figures 6C,D. Accordingly, the proposed scGNGI method helps identify different cell types more accurately in real and simulated data.

### Marker Genes Analysis

In biology, the marker genes define the cell populations and reveal substantial cell markers to distinguish different cell types. Apart from improving the overall imputation accuracy, the proposed imputation method needs to capture the dependence relationship between marker genes. For the real scRNA-seq data (Cell Type dataset), we identified marker genes (KLF4, NANOG, SOX2, CD9, CDH11, EFNA2, and PRSS50) by searching markers of diverse cell types in CellMarker databases (Zhang et al., 2019). Given that these scRNA-seq data were obtained from complex biological systems, there are many non-linear gene–gene dependencies from complex and multi-cell type samples. It is also much more difficult to capture the non-linear relationships between genes. Furthermore, a marker gene can be used to delineate between taxonomic lineages. Here, we examine the non-linear relationships between the marker gene pairs in the Cell Type data imputed by different methods and explore the imputation performance of different methods for the marker genes.

#### Recovery of Non-linear Gene–Gene Relationships

**FIGURE 7:**
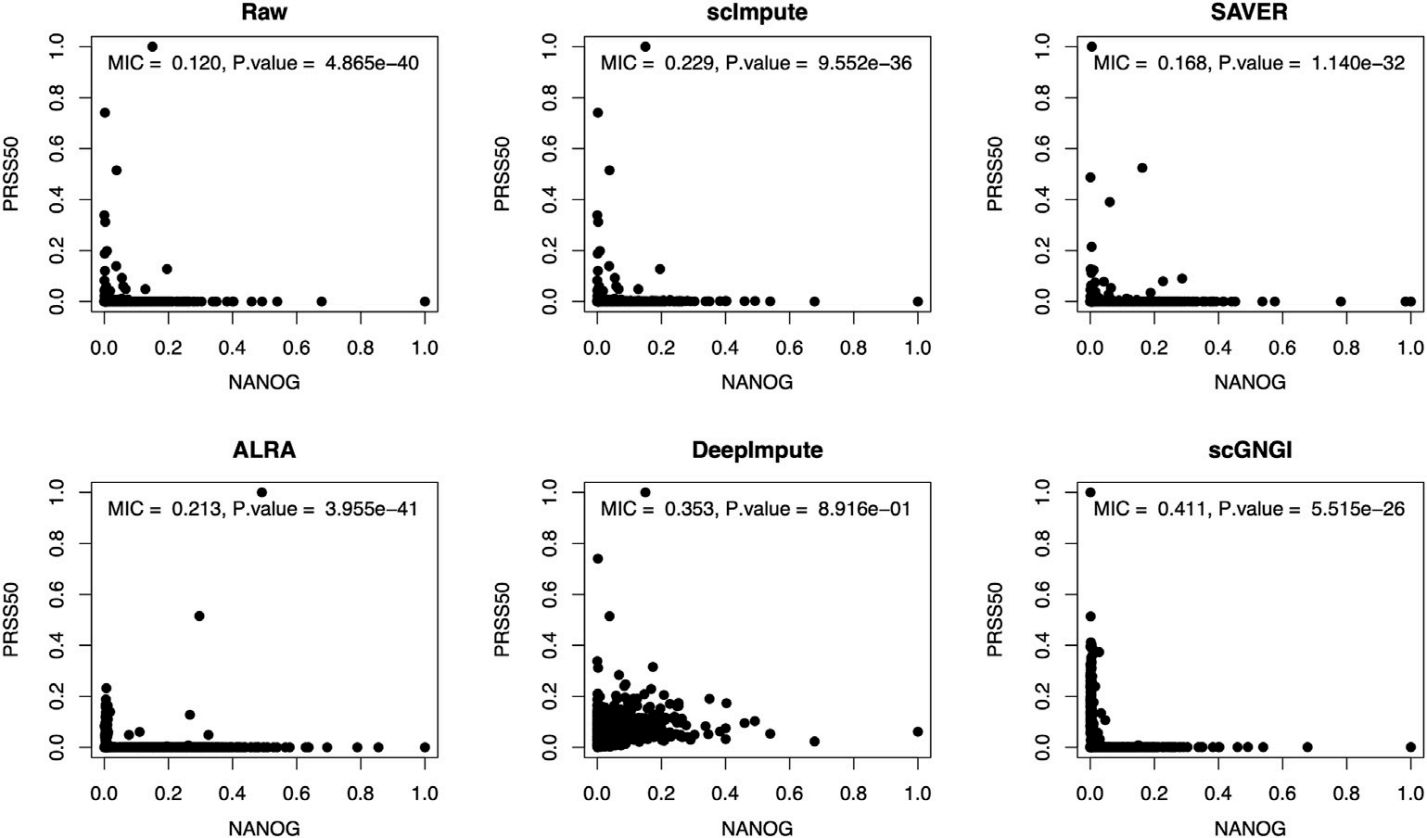
Scatter plots of the expression levels between marker genes (NANOG and PRSS50) in the raw and imputed data by using different methods. On the top, the corresponding maximal information coefficient (MIC) and *p* value are shown for gene expression values. The two-sided Wilcoxon rank-sum test is used for *p* values. Each dot represents a cell.

To explore the non-linear relationships between marker genes in the imputed data, we use the maximal information coefficient (MIC) (Reshef et al., 2011) to evaluate the recovery of non-linear gene–gene relationships. In statistics, the MIC is a measure of non-linear association between two variables, which belongs to the maximal information-based non-parametric exploration (MINE). Here, the MIC is defined to measure the non-linear dependence structure between estimated gene pairs. As shown in Figure 7, the proposed scGNGI obtains the highest MIC value ( $MIC=0.411,\;P.value=5.515\times 10^{-26}$ ) for the marker gene pair (NANOG and PRSS50), compared to scImpute, SAVER, ALRA, and DeepImpute. Additionally, Supplementary Figure S9 shows that the non-linear recovering performance by our method outperforms scImpute, SAVER, and DeepImpute for the marker gene pair (EFNA2 and PRSS50). While the MIC value by the proposed scGNGI ( $MIC=0.374,\;P.value=4.439\times 10^{-06}$ ) is slightly lower than ALRA ( $MIC=0.411,\;P.value=6.064\times 10^{-70}$ ) in Supplementary Figure S9. For other marker gene pairs (SOX2 and PRSS50, CDH11 and PRSS50), our method still better recovers the non-linear relationships between marker genes, as shown in Supplementary Figures S10, S11. Although our method fails to recover the strong non-linear relationships ( MIC>0.8 ) in the four marker gene pairs, it can still recover the general and weak non-linear relationships between marker genes ( MIC>0.3 ). However, as shown in Figure 7 and Supplementary Figures S9–S11, not including ALRA in Supplementary Figure S9, the MIC values between imputed marker genes by the other methods fail to exceed 0.3. This shows that the proposed scGNGI method can recover the non-linear relationships between marker genes.

#### Imputation Performance of Different Methods for Marker Genes

**FIGURE 8:**
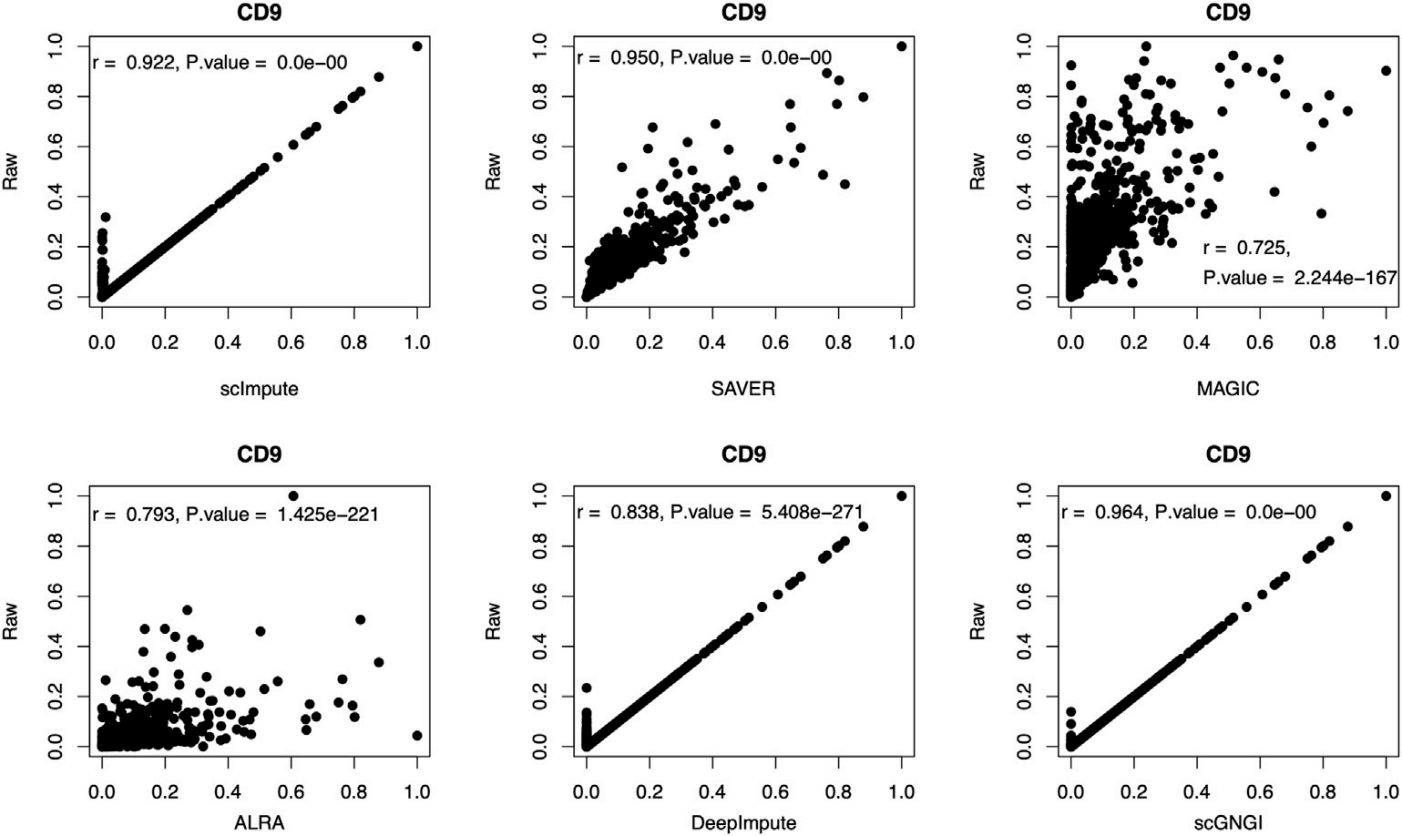
Scatter plots of the expression level for the marker gene (CD9) in the raw and imputed data by using different methods. On the top, the corresponding correlation coefficient (r) and *p* value are shown for gene expression values. The two-sided correlation test is used for *p* values. Each dot represents a cell.

To show the imputation performance of different methods for the marker genes, we computed the correlation coefficient between marker genes in the raw and imputed data by different methods. As shown in Figure 8, for the marker gene (CD9), we obtain the highest correlation coefficient ( r=0.964 ) performed by the proposed scGNGI method, compared to scImpute, SAVER, MAGIC, ALRA, and DeepImpute. This shows that our method can maintain a better correlation with the expression level of the raw marker gene (CD9). Furthermore, for another marker gene (NANOG), we find that the proposed scGNGI can obtain similar results with marker gene (CD9), as shown in Supplementary Figure S12. Accordingly, our method can better impute expression values of marker genes in the real scRNA-seq data.

### Coefficient of Variation for the Gene Expression

Missing gene expression in a cell affects the scRNA-seq data analysis of the corresponding cell type.

**FIGURE 9:**
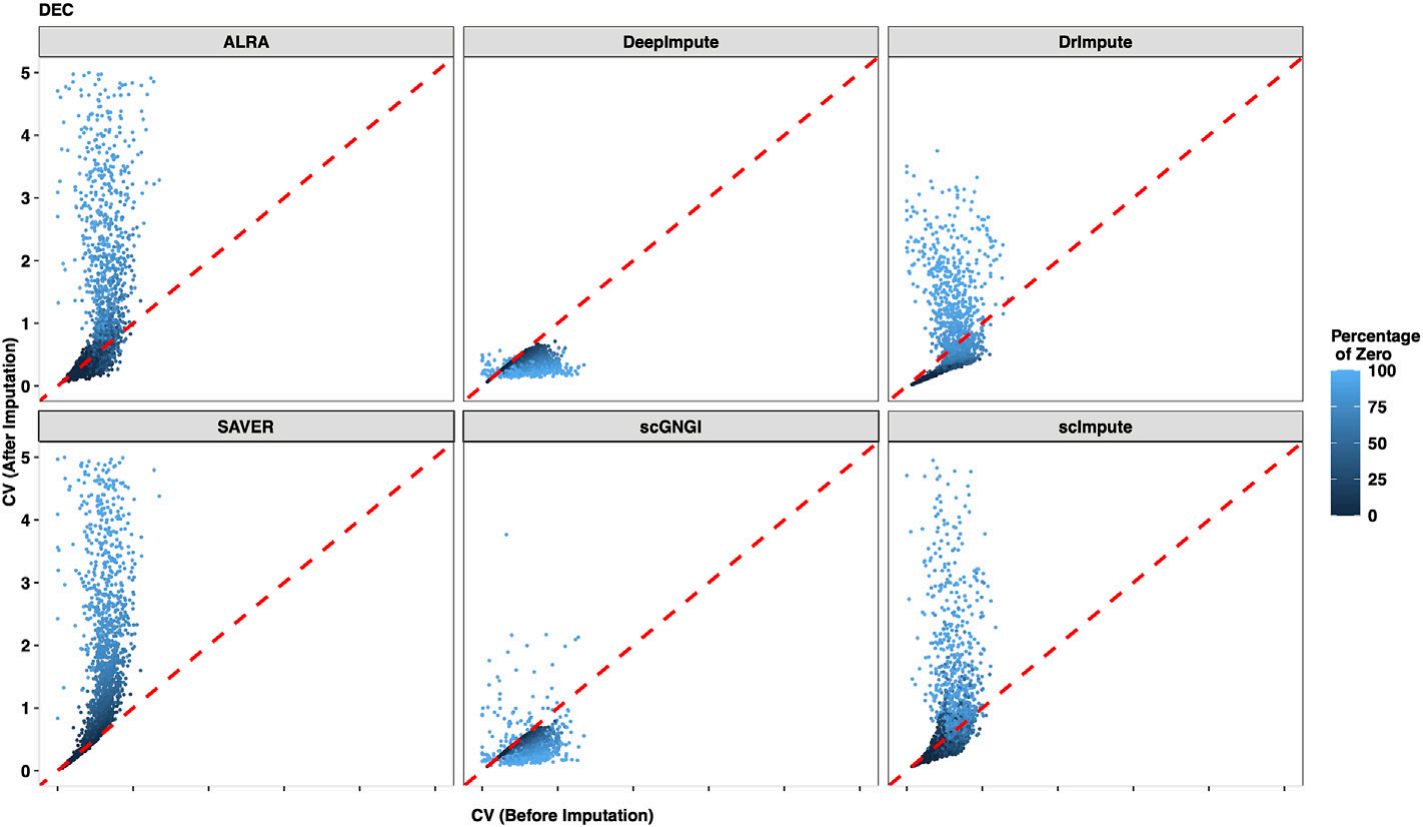
Gene expression variation between the raw data and the imputed data for DEC cells in the Cell Type dataset. The coefficient of variation (CV) is computed for each gene in all DEC cells after imputation (*y*-axis) by using different methods, and the *x*-axis represents the CV of non-zero cells before imputation. Each dot represents a gene, and the mean level of the non-zero values is distinguished by its color.

It is necessary to evaluate the imputation of all missing values within individual cell types. To quantify the recovering performance of the mean gene expression within individual cell types, we computed the coefficient of variation (CV) to represent the gene expression variability between cells. For each cell type, we compared the CV of non-zero values (before imputation) with the CV of the imputed values (after imputation) between cells. As shown in Figure 9, two CV values (before and after imputation) from DEC cells are constructed in Cell Type data, and the gradually changing colors represent the different zero proportions (non-zero mean gene expression levels). Generally, if the dropout events result in zero values of the gene expression, the two CV values are expected to be similar before and after imputation. This is because the distributions of non-zero values and imputed values are consistent before and after imputation. Conversely, if the low gene expression leads to these zero values, the CV value after imputation is expected to be higher than the CV value of non-zero gene expression. This is because the gene expression values of non-zero values are usually higher than the imputed data before and after imputation. Accordingly, the CV value after imputation is either higher than or equal to the CV before imputation. In Figure 9, we find that our results satisfy the aforementioned explanation, where two CV values of most genes from the DEC cells are similar before and after imputation in Cell Type data. In addition, the CV values of most imputed genes are higher for DEC cells imputed by ALRA, DrImpute, SAVER, and scImpute. This suggests that ALRA, DrImpute, SAVER, and scImpute regard non-dropout events as the source of most zero values. For almost all genes, we can obtain the smaller CV values of imputed data by DeepImpute, which shows that DeepImpute reduces the gene expression variability within DEC cells after imputation.

As shown in Supplementary Figures S13–S18, we also examine other cell types (EC, H1, H9, HFF, NPC, and TB) in the Cell Type dataset. For the EC, H1, H9, HFF, NPC, and TB cells, we still find similar results with DEC cells. Especially, for the five cell types in Simulated Data 1, and four cell types Simulated Data 2, these results show similar patterns with the Cell Type data, as shown in Supplementary Figures S19–S23, and Supplementary Figures S24–S27. More reasonably, the zero values are viewed as the dropout events for the imputation of the scRNA-seq data. The aforementioned results suggest that the proposed scGNGI regards the dropout events as the main source of missing values to impute the scRNA-seq data. Generally, our method can preserve gene expression variability within each cell type while imputing the expression values of scRNA-seq data.

## Discussion

Single-cell RNA-sequencing (scRNA-seq) technologies have improved the measurements of gene expression in individual cells. However, various technical noises complicate the analysis of cell patterns, which leads to false zero values (missing gene expression values) in the scRNA-seq data. It is still a challenge to recover missing gene expression values more effectively in the scRNA-seq data. We use Gauss–Newton imputation to impute the missing values in scRNA-seq expression matrices. The experimental results have shown that the proposed method can effectively impute missing values.

In detail, the experimental results of the mask data show that the missing values imputed by our method are closer to the real values (ground truth). Since our method considers the cell heterogeneity by regarding the number of cell types as an optimization constraint, we make the imputed data maintain the characteristics of the original data to a greater extent. Compared with other imputation methods, it is more reasonable to impute missing values by using the proposed methods. Furthermore, the results of cell type visualization show that the obtained high-quality data using the proposed method make various cell subpopulations more separable. The cluster results show that our method can improve the clustering accuracy more effectively. In biology, the marker genes define the cell populations. The recovered marker gene expressions have shown that the proposed method helps distinguish different cell types. To recover mean gene expression within individual cell types, we explore the coefficient of variation (CV) between cells before and after imputation. The result of CV shows that the gene expression variability can be better preserved within each cell type using the proposed scGNGI method. Given of the limitations of the scGNGI model, our method does fail to achieve an excellent recovery performance for a large number of missing values, such as more than 70% of the missing items in the scRNA-seq data. To recover a large number of missing values, we will consider applying prior information from bulk data to the proposed scGNGI. In addition, we can consider improving the proposed scGNGI method for other biological data, such as sequence data. In the future, the improved scGNGI will be applied to some features generated by some tools, such as BioSeq-Analysis2.0 (Liu, et al., 2019) and BioSeq-BLM (Li, et al., 2021), which would improve the performance of the current method.

## Conclusion

The single-cell RNA sequencing technology enhances the characterization of thousands of individual cells. Recovering missing gene expression values improves the analysis of cell patterns at the single-cell level. Here, we present a novel imputation method, i.e., scGNGI, to impute the missing gene expression values in the scRNA-seq data by combining the low-rank matrix completion with the potential cell heterogeneity. The experimental results show that scGNGI effectively imputes the missing values of gene expression and improves the low-dimensional representation. Furthermore, the cells clustering and identifying cell types are also enhanced in the imputed data. It is easier to recover the non-linear relationships between imputed marker genes. Especially, the results of the gene expression variability among cells suggest that the proposed scGNGI views the dropout events as the main source of zero values to estimate the missing gene expression more reasonably. In general, our method is more helpful for exploring the complex biological system in scRNA-seq data and improving the cancer-related disease therapy and precision medicine.

## Summary

The single-cell RNA sequencing technology has improved the analysis of individual cells in transcriptome studies. The proposed scGNGI is an effective imputation method to impute the scRNA-seq data by using the low-rank matrix completion with the potential cell heterogeneity. scGNGI improves the low-dimensional representation and the identification of cell types in the low-quality scRNA-req data. In addition, scGNGI facilitates the clustering accuracy of cells and the non-linear relationships between marker genes. The results of gene expression variability show that scGNGI models the zero values of gene expression caused by the dropout events. Specifically, our methods help explore the complex biological system and improve the analysis about cancer-related diseases in scRNA-seq data. In the future, we will consider imputing a large of missing values in a scRNA-seq matrix by improving the proposed scGNGI.

## Data Availability

The source code used to replicate analysis, including synthetic and real datasets, is available at the following link: https://github.com/linxi159/scGNGI. Further inquiries can be directed to the corresponding author.
