# Supplementary material for: Missing Value Imputation With Low-Rank Matrix Completion in Single-Cell RNA-Seq Data by Considering Cell Heterogeneity
Source: Front Genet. 2022 Jul 14;13:952649. doi: 10.3389/fgene.2022.952649 (PMC9329700; doi:10.3389/fgene.2022.952649)
Supplement: Supplementary file 1 [file DataSheet1.docx]

Supplementary Material

# Supplementary Figures


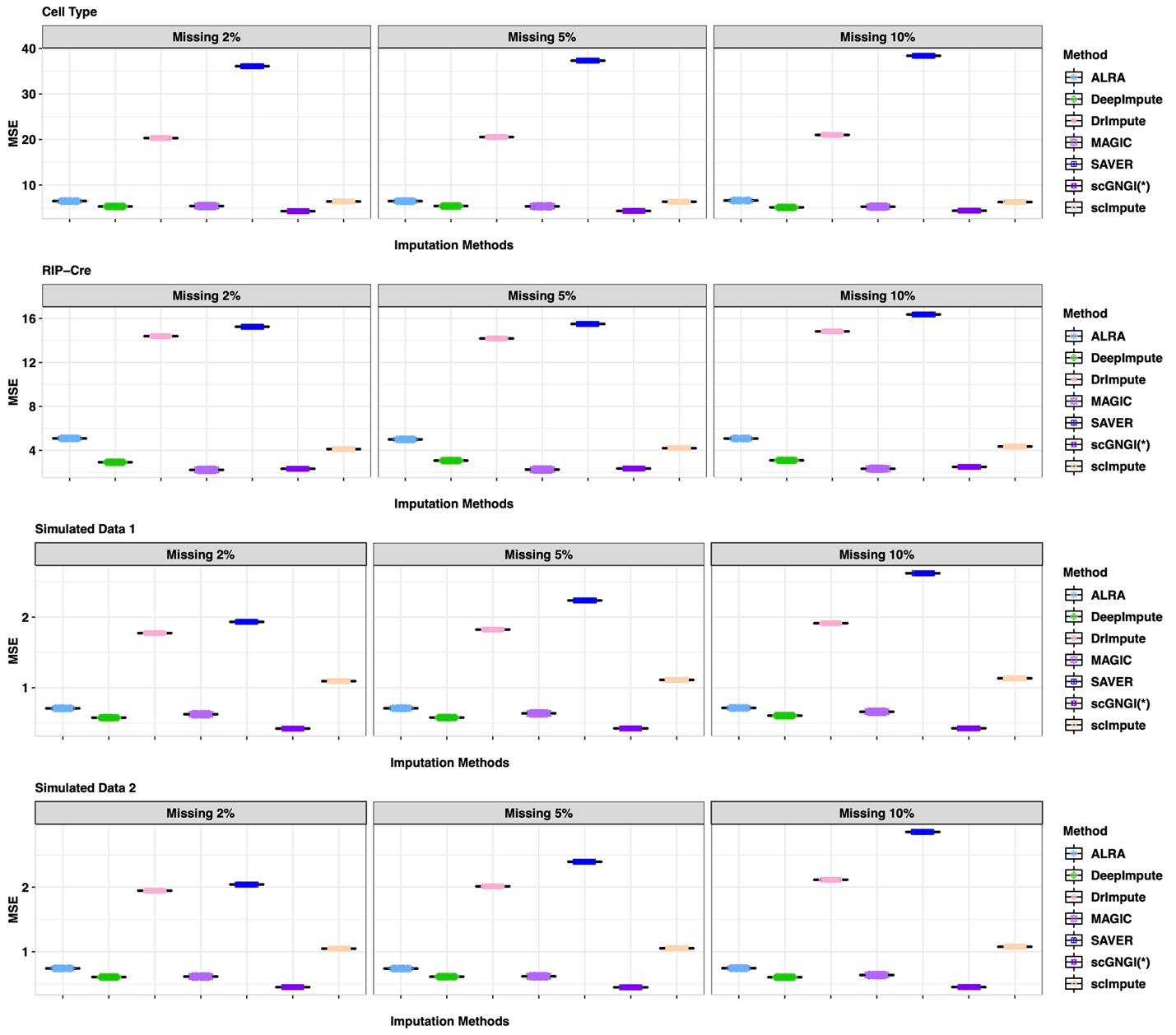


**Supplementary Figure S1.** Mean squared error between the masked truth and imputed values by different methods in the scRNA-seq data masking experiments. Rows represent four different datasets (Cell Type, RIP-Cre, Simulated Data 1, and Simulated Data 2). Columns represent three different masking percentages (2%, 5% and 10%). Boxplots represent the mean squared error values from 60 masking replicates, where the mean squared error of each cell is calculated in turn and then are plotted across cells.


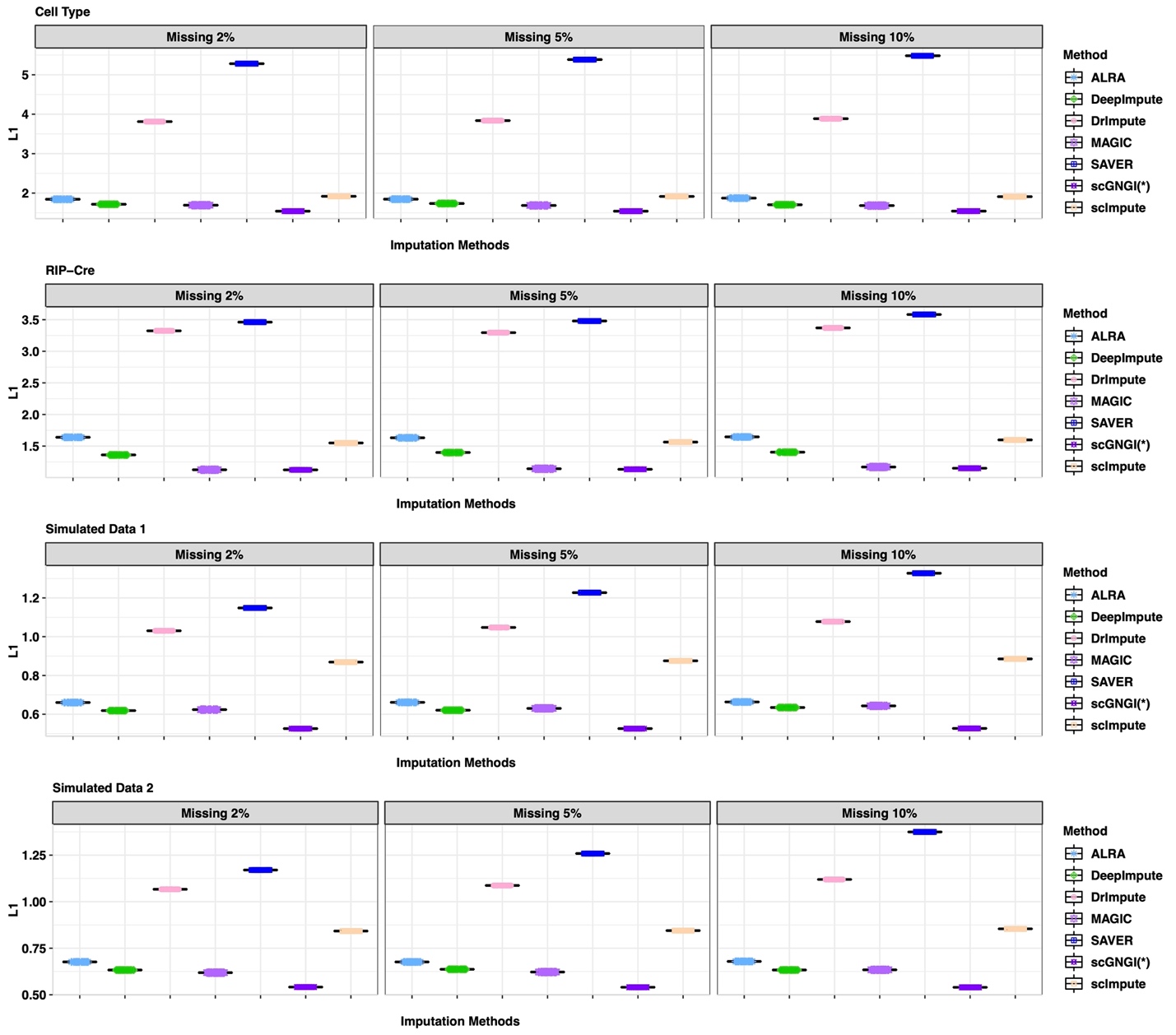


**Supplementary Figure S2.** L1 norm between the masked truth and imputed values by different methods in the scRNA-seq data masking experiments. Rows represent four different datasets (Cell Type, RIP-Cre, Simulated Data 1, and Simulated Data 2). Columns represent three different masking percentages (2%, 5%, and 10%). Boxplots represent the L1 values from 60 masking replicates, where the L1 of each cell is calculated in turn and the mean L1 is plotted across cells.


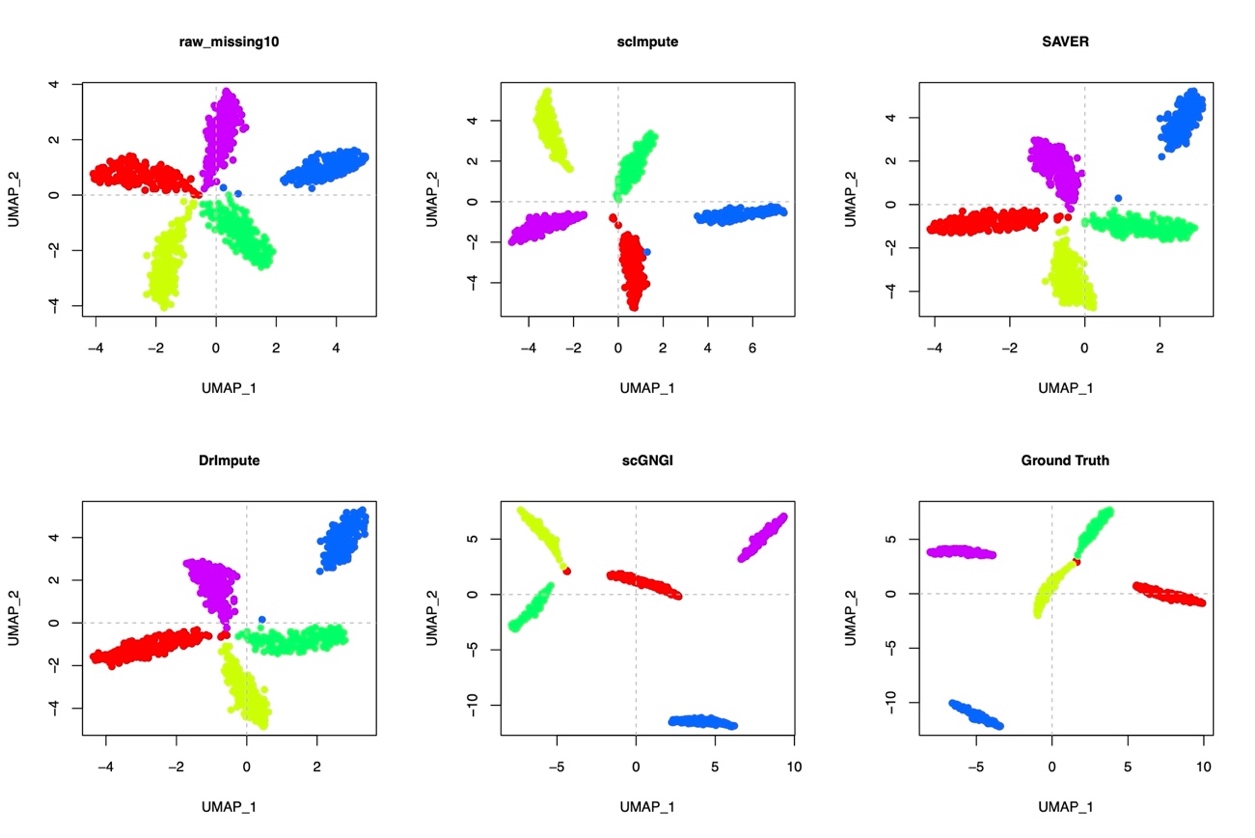


**Supplementary Figure S3.** Imputation performance on Simulated Data 1 with 5 known cell types. Visualization of cells by the first two UMAP components on the raw data, missing data, and imputed ones by different methods. Each dot is a single cell, and different colors represent different cell types.


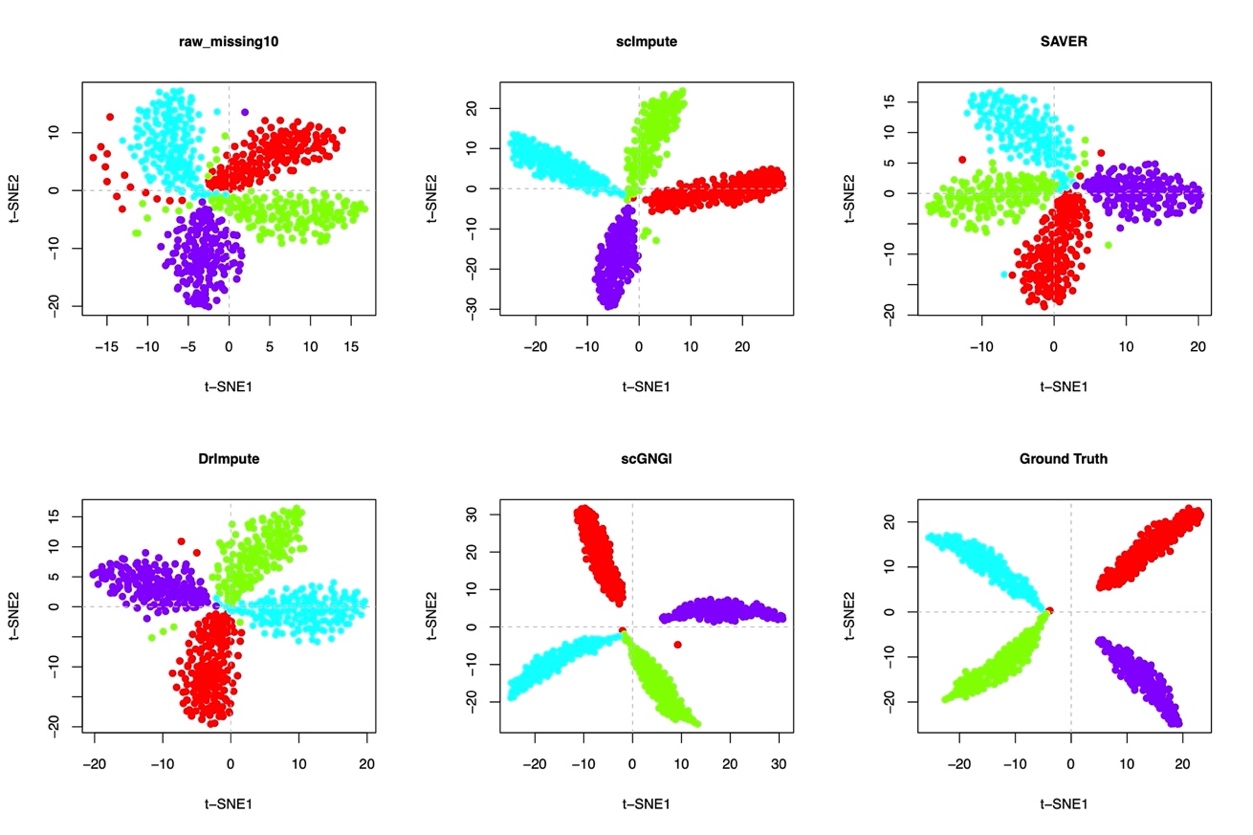


**Supplementary Figure S4.** Imputation performance on Simulated Data 2 with 4 known cell types. Visualization of cells by the first two t-SNE components on the raw data, missing data, and imputed ones by different methods. Each dot is a single cell, and different colors represent different cell types.


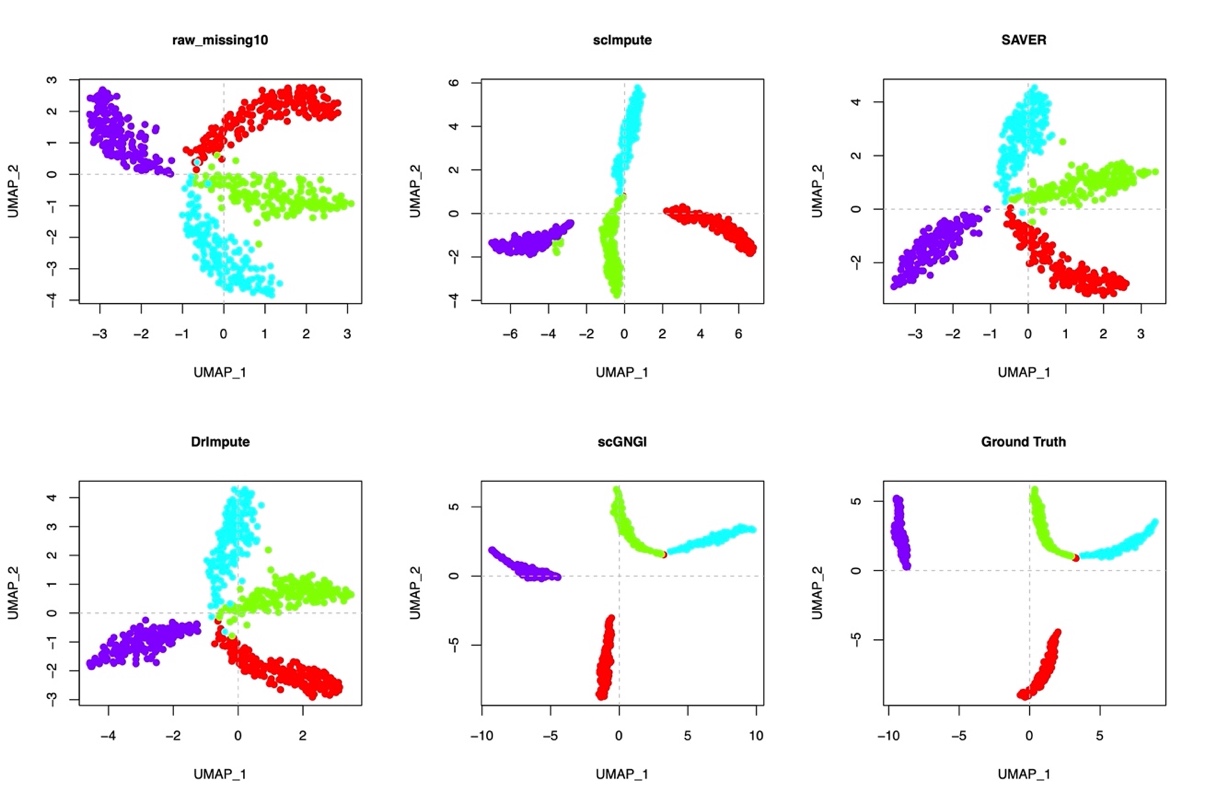


**Supplementary Figure S5.** Imputation performance on Simulated Data 2 with 4 known cell types. Visualization of cells by the first two UMAP components on the raw data, missing data, and imputed ones by different methods. Each dot is a single cell, and different colors represent different cell types.


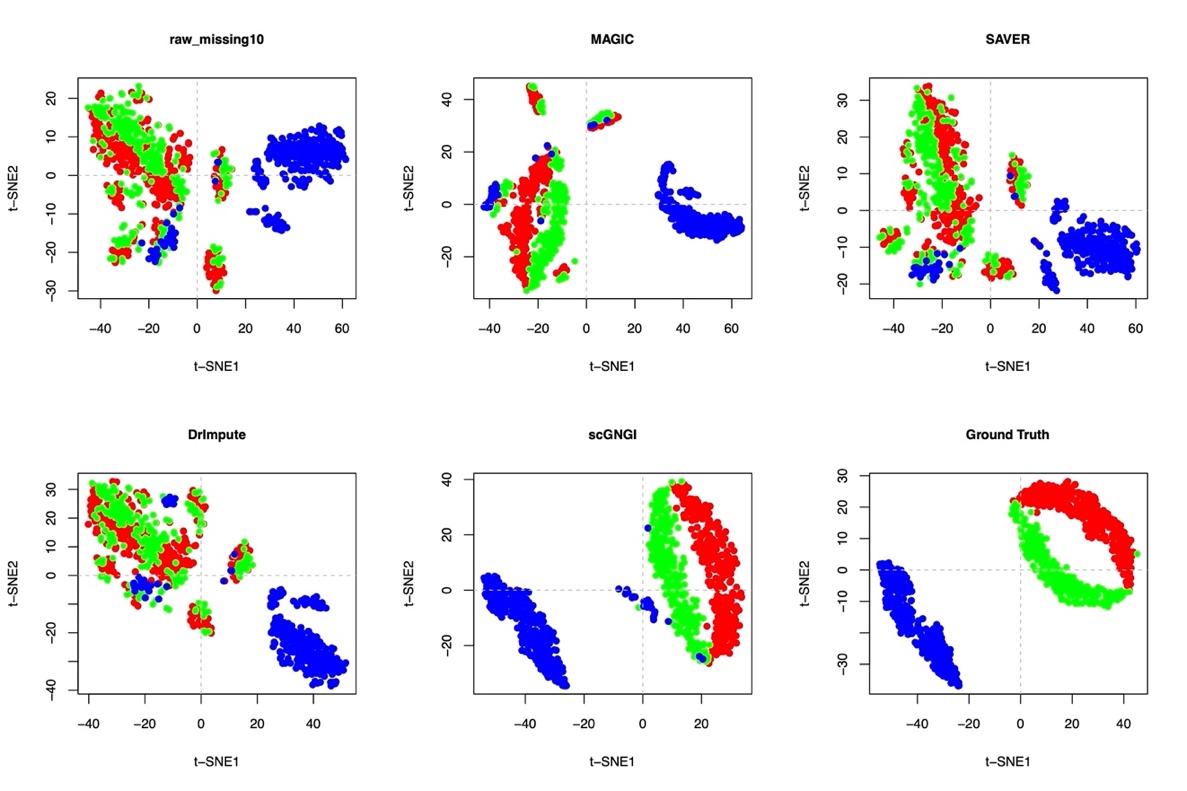


**Supplementary Figure S6.** Imputation performance on Simulated Data 3 with 3 known cell types. Visualization of cells by the first two UMAP components on the raw data, missing data (masking percentage 10%), and imputed ones by different methods. Each dot is a single cell, and different colors represent different cell types.


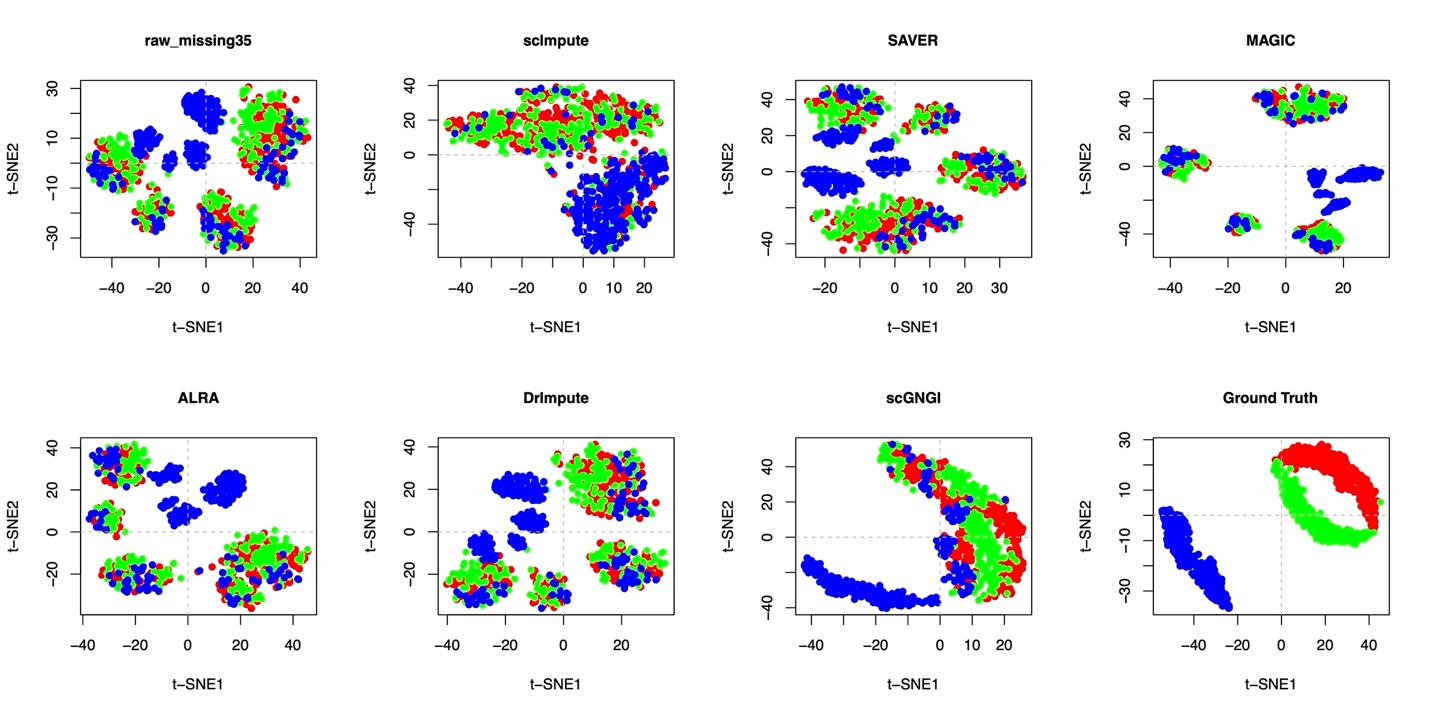


**Supplementary Figure S7.** Imputation performance on Simulated Data 3 with 3 known cell types. Visualization of cells by the first two t-SNE components on the raw data, missing data (masking percentage 35%), and imputed ones by different methods. Each dot is a single cell, and different colors represent different cell types.


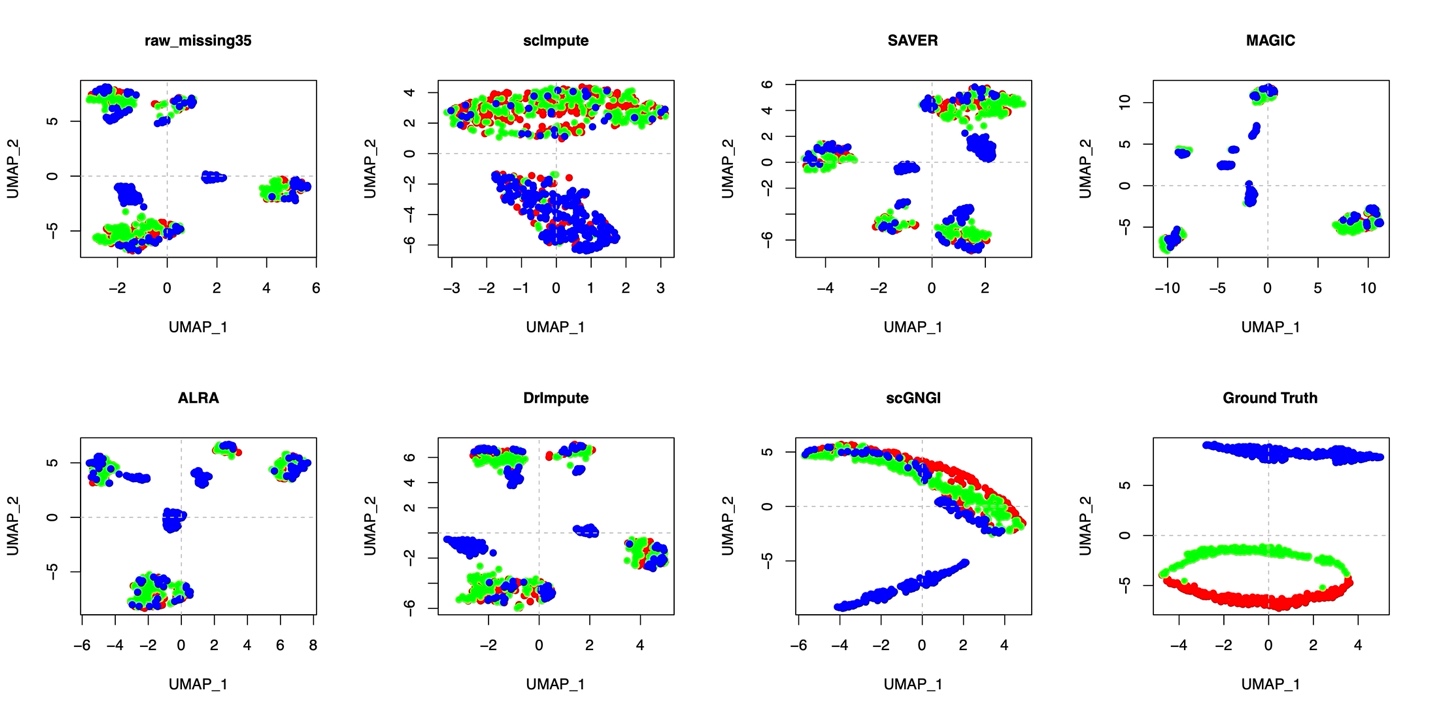


**Supplementary Figure S8.** Imputation performance on Simulated Data 3 with 3 known cell types. Visualization of cells by the first two UMAP components on the raw data, missing data (masking percentage 35%), and imputed ones by different methods. Each dot is a single cell, and different colors represent different cell types.


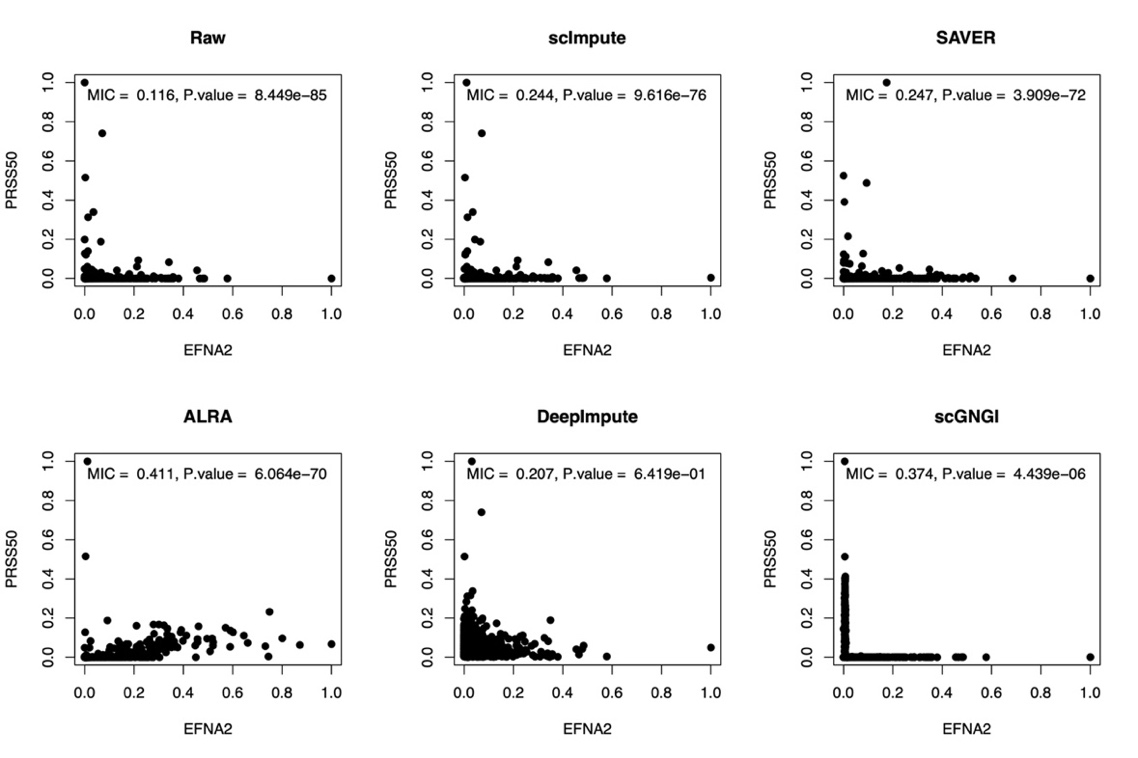


**Supplementary Figure S9.** Scatter plots of the expression level between marker genes (EFNA2 and PRSS50) in the raw and imputed data by different methods. On the top, the corresponding maximal information coefficient (MIC) and P.value are shown for gene expression values. The two-sided Wilcoxon rank-sum test is used for P.values. Each dot represents a cell.


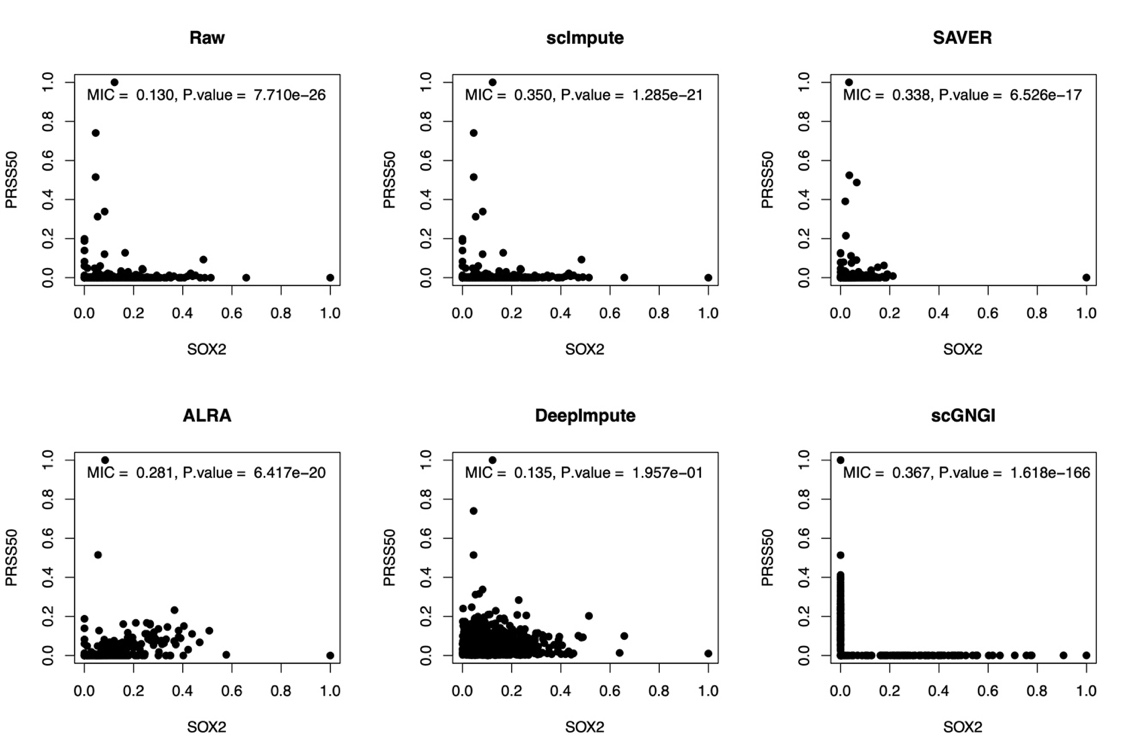


**Supplementary Figure S10.** Scatter plots of the expression level between marker genes (SOX2 and PRSS50) in the raw and imputed data by different methods. On the top, the corresponding maximal information coefficient (MIC) and P.value are shown for gene expression values. The two-sided Wilcoxon rank-sum test is used for P.values. Each dot represents a cell.


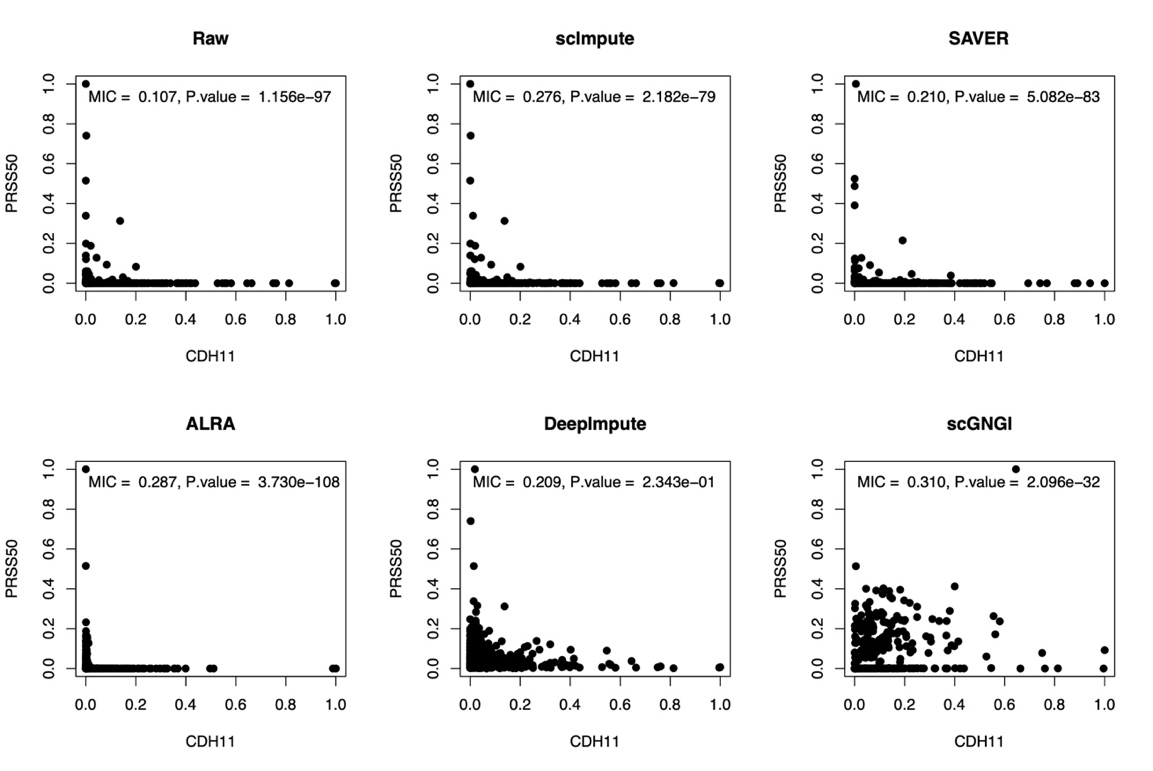


**Supplementary Figure S11.** Scatter plots of the expression level between marker genes (CDH11 and PRSS50) in the raw and imputed data by different methods. On the top, the corresponding maximal information coefficient (MIC) and P.value are shown for gene expression values. The two-sided Wilcoxon rank-sum test is used for P.values. Each dot represents a cell.


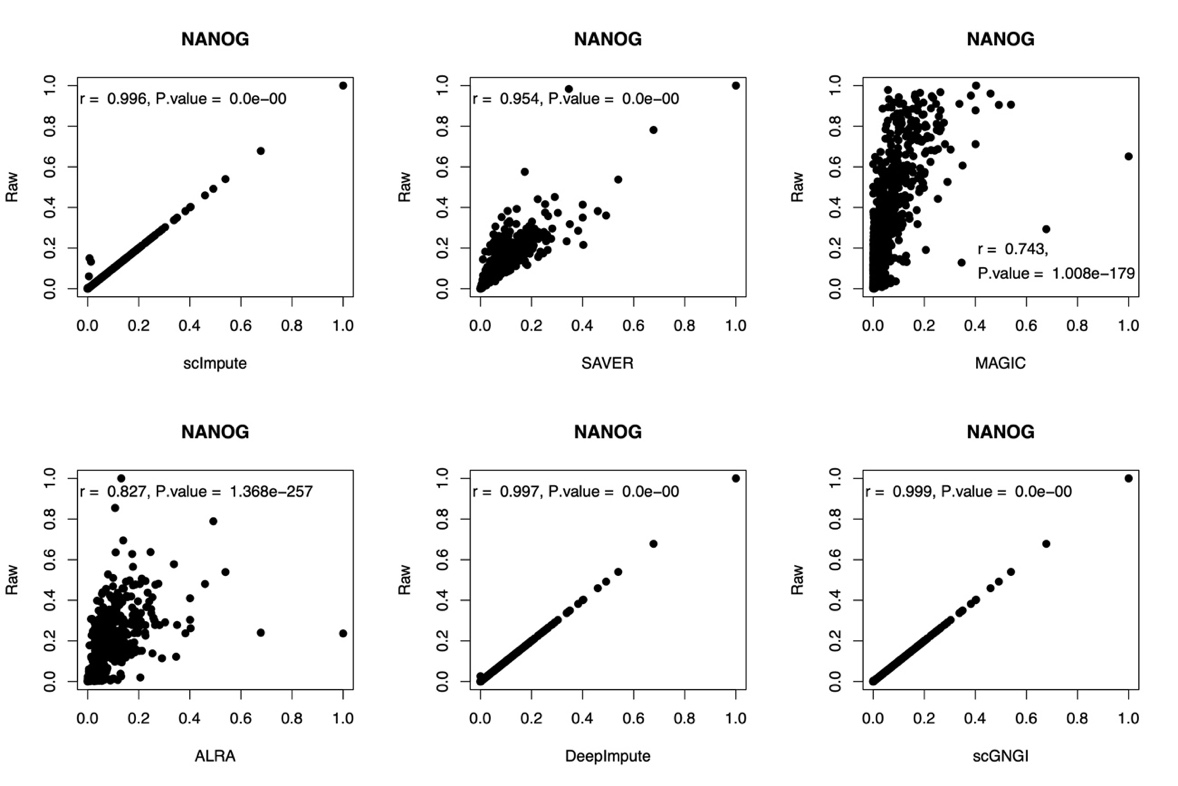


**Supplementary Figure S12.** Scatter plots of the expression level for the marker gene (NANOG) in the raw and imputed data by different methods. On the top, the corresponding correlation coefficient (r) and P.value are shown for gene expression values. The two-sided correlation test is used for P.values. Each dot represents a cell.


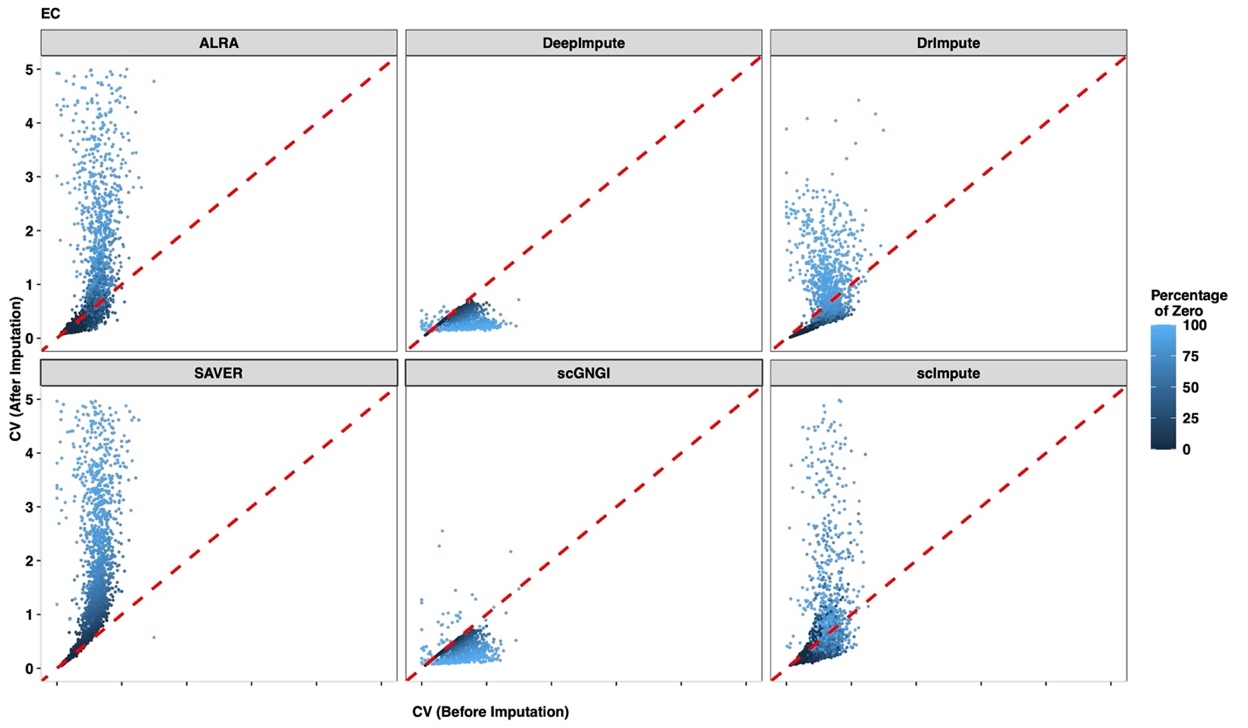


**Supplementary Figure S13.** Gene expression variation between the raw data and the imputed data for EC cells in the Cell Type dataset. The coefficient of variation (CV) is computed for each gene in all EC cells after imputation (y-axis) by different methods, and the x-axis represents the CV of non-zero cells before imputation. Each dot represents a gene, and the mean level of non-zero values is distinguished by its color.


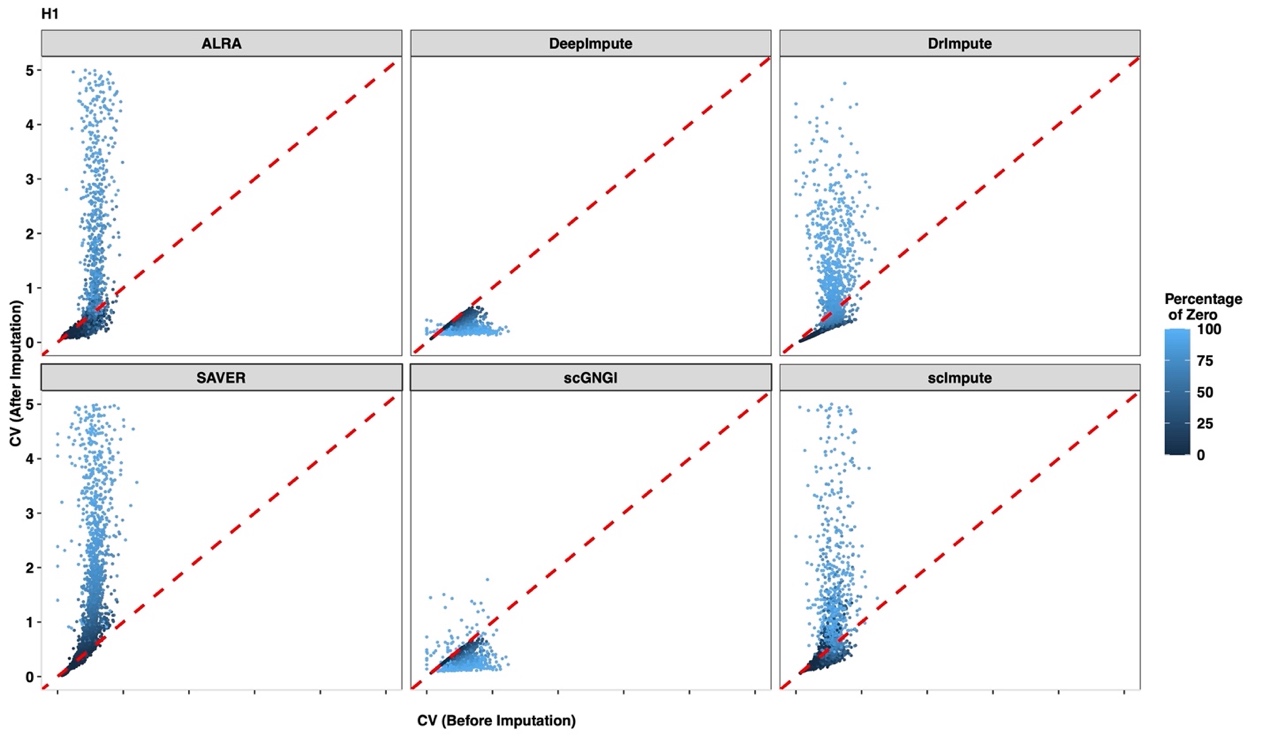


**Supplementary Figure S14.** Gene expression variation between the raw data and the imputed data for H1 cells in the Cell Type dataset. The coefficient of variation (CV) is computed for each gene in all H1 cells after imputation (y-axis) by different methods, and the x-axis represents the CV of non-zero cells before imputation. Each dot represents a gene, and the mean level of non-zero values is distinguished by its color.


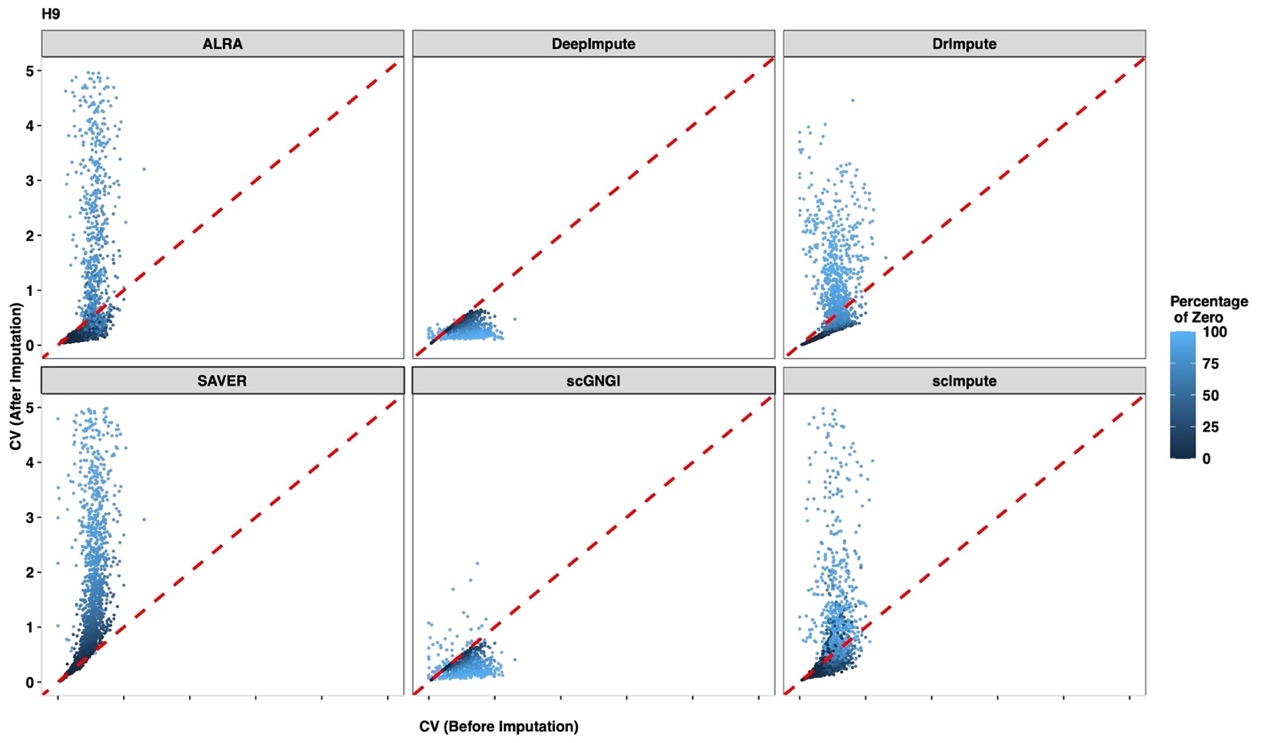


**Supplementary Figure S15.** Gene expression variation between the raw data and the imputed data for H9 cells in the Cell Type dataset. The coefficient of variation (CV) is computed for each gene in all H9 cells after imputation (y-axis) by different methods, and the x-axis represents the CV of non-zero cells before imputation. Each dot represents a gene, and the mean level of non-zero values is distinguished by its color.


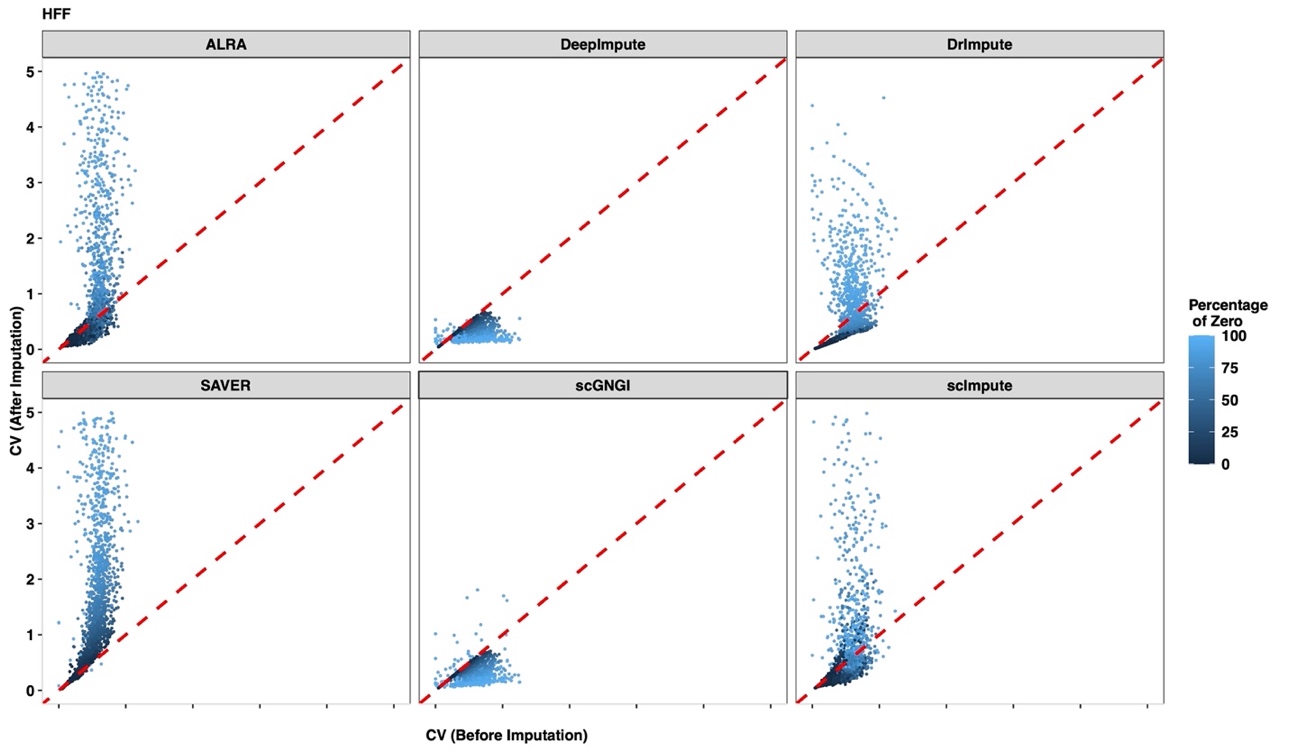


**Supplementary Figure S16.** Gene expression variation between the raw data and the imputed data for HFF cells in the Cell Type dataset. The coefficient of variation (CV) is computed for each gene in all HFF cells after imputation (y-axis) by different methods, and the x-axis represents the CV of non-zero cells before imputation. Each dot represents a gene, and the mean level of non-zero values is distinguished by its color.


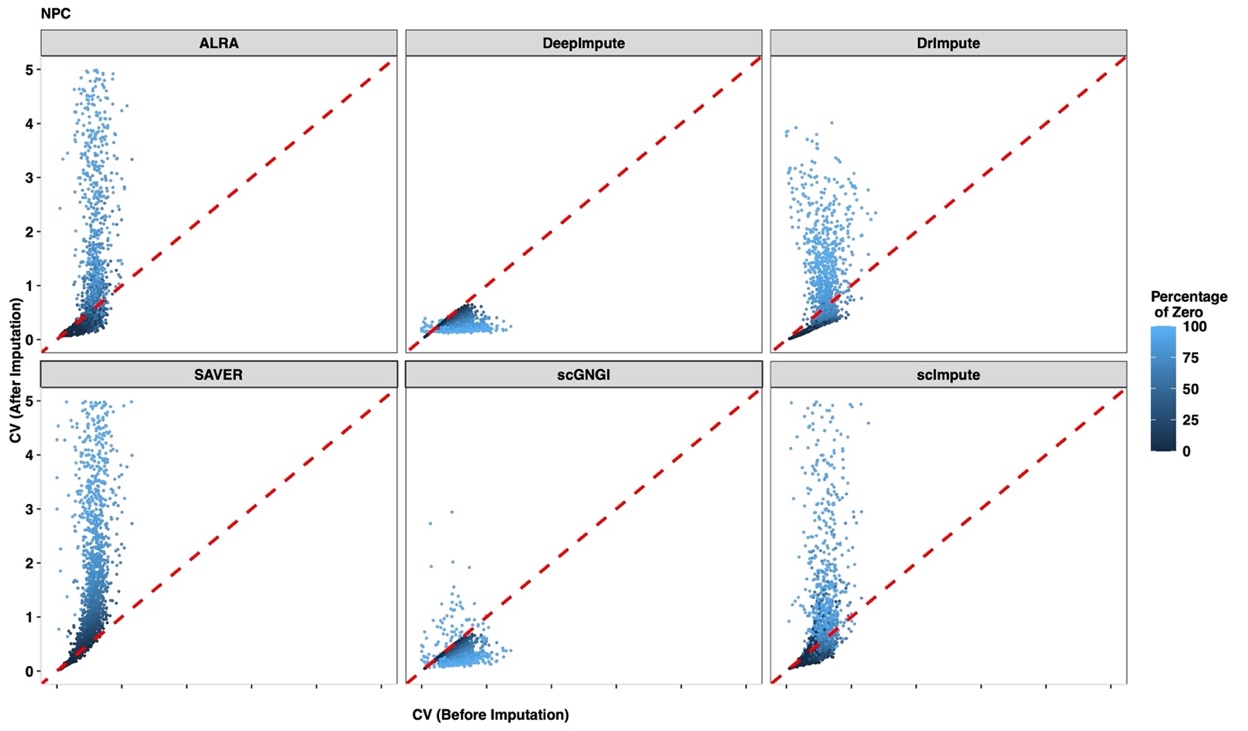


**Supplementary Figure S17.** Gene expression variation between the raw data and the imputed data for NPC cells in the Cell Type dataset. The coefficient of variation (CV) is computed for each gene in all NPC cells after imputation (y-axis) by different methods, and the x-axis represents the CV of non-zero cells before imputation. Each dot represents a gene, and the mean level of non-zero values is distinguished by its color.


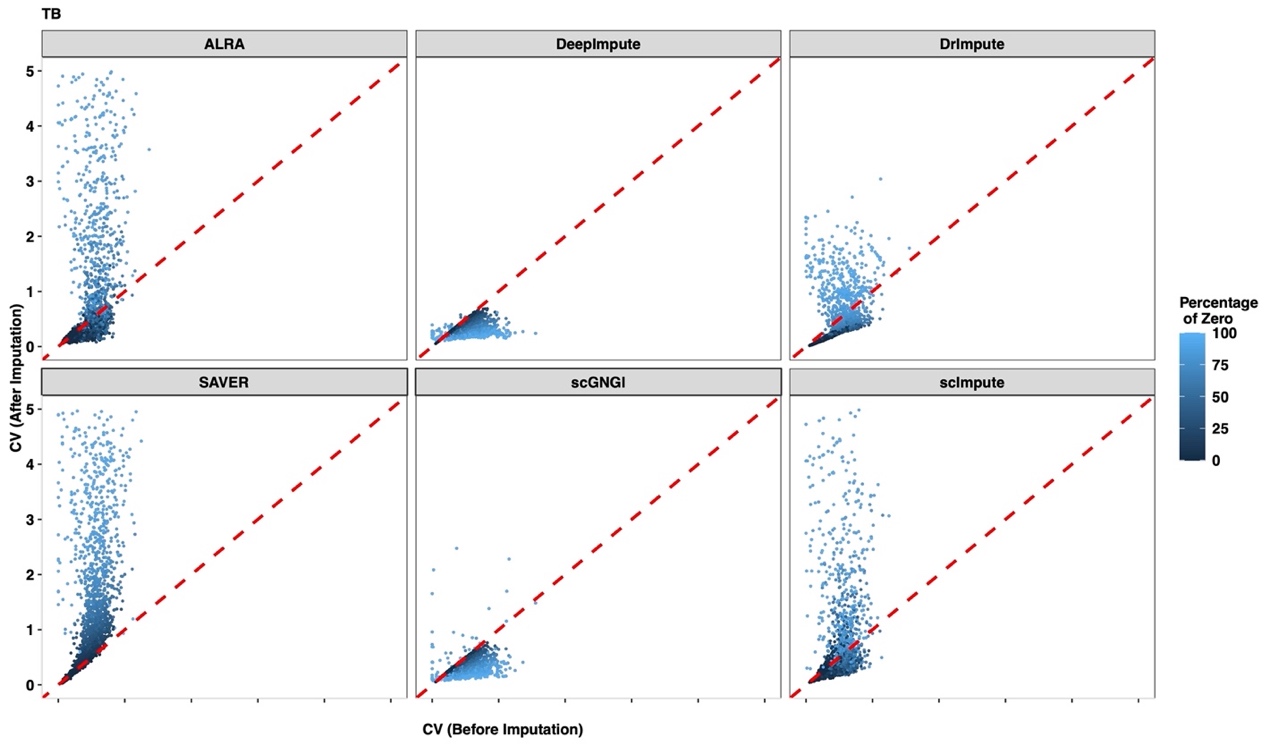


**Supplementary Figure S18.** Gene expression variation between the raw data and the imputed data for TB cells in the Cell Type dataset. The coefficient of variation (CV) is computed for each gene in all TB cells after imputation (y-axis) by different methods, and the x-axis represents the CV of non-zero cells before imputation. Each dot represents a gene, and the mean level of non-zero values is distinguished by its color.


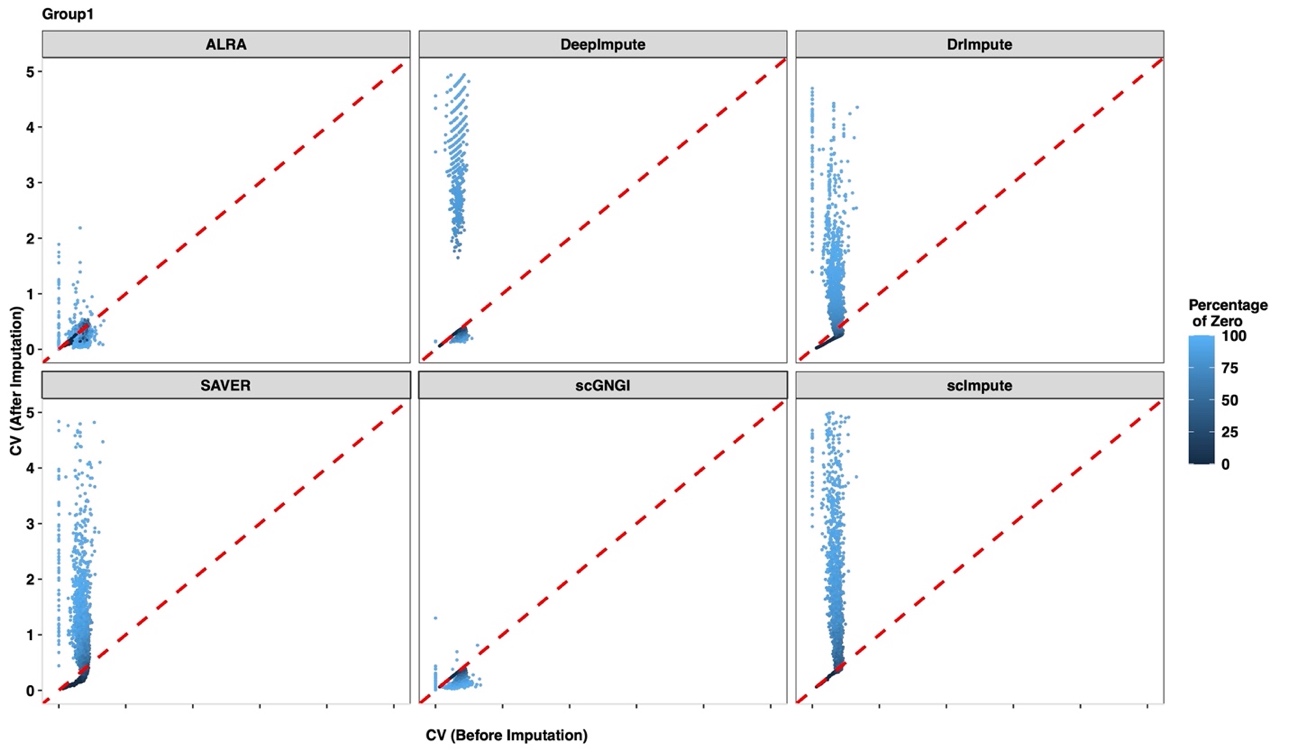


**Supplementary Figure S19.** Gene expression variation between the raw data and the imputed data for Group1 cells in the Simulated Data 1 dataset. The coefficient of variation (CV) is computed for each gene in all Group1 cells after imputation (y-axis) by different methods, and the x-axis represents the CV of non-zero cells before imputation. Each dot represents a gene, and the mean level of non-zero values is distinguished by its color.


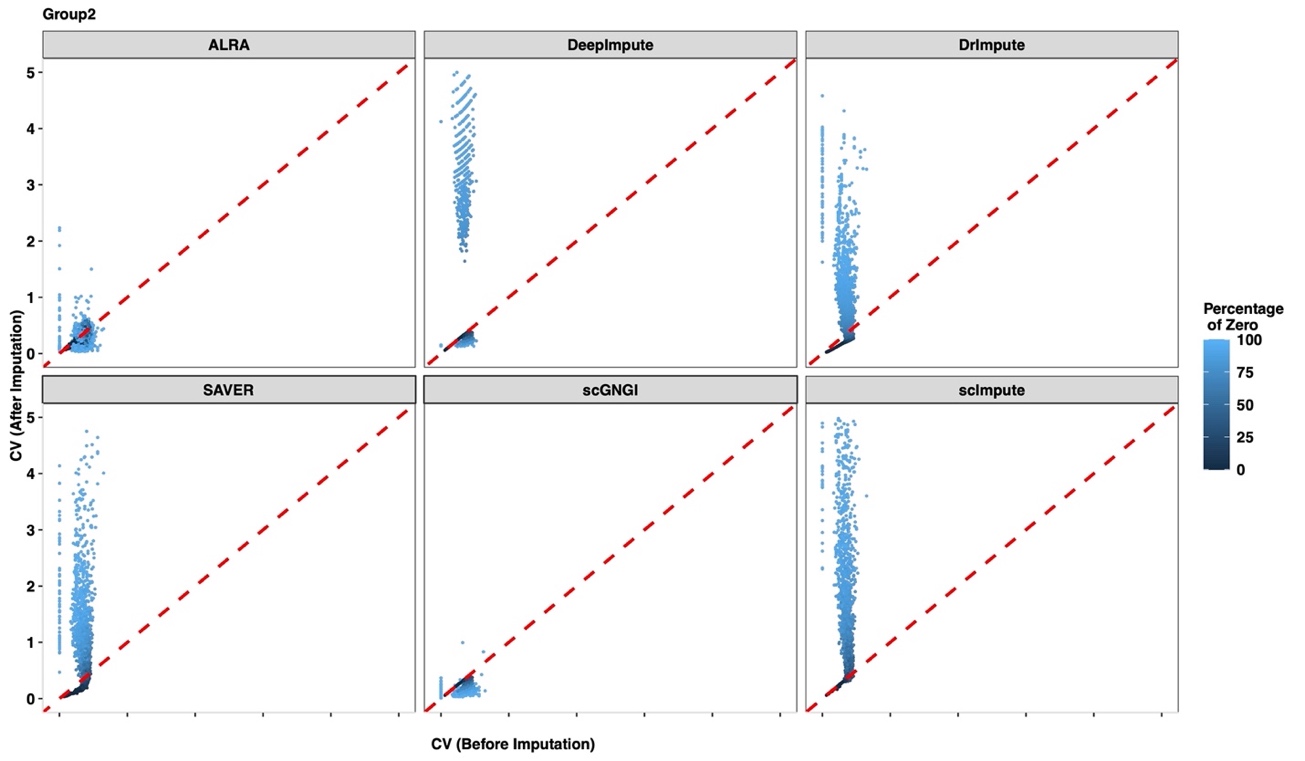


**Supplementary Figure S20.** Gene expression variation between the raw data and the imputed data for Group2 cells in the Simulated Data 1 dataset. The coefficient of variation (CV) is computed for each gene in all Group2 cells after imputation (y-axis) by different methods, and the x-axis represents the CV of non-zero cells before imputation. Each dot represents a gene, and the mean level of non-zero values is distinguished by its color.


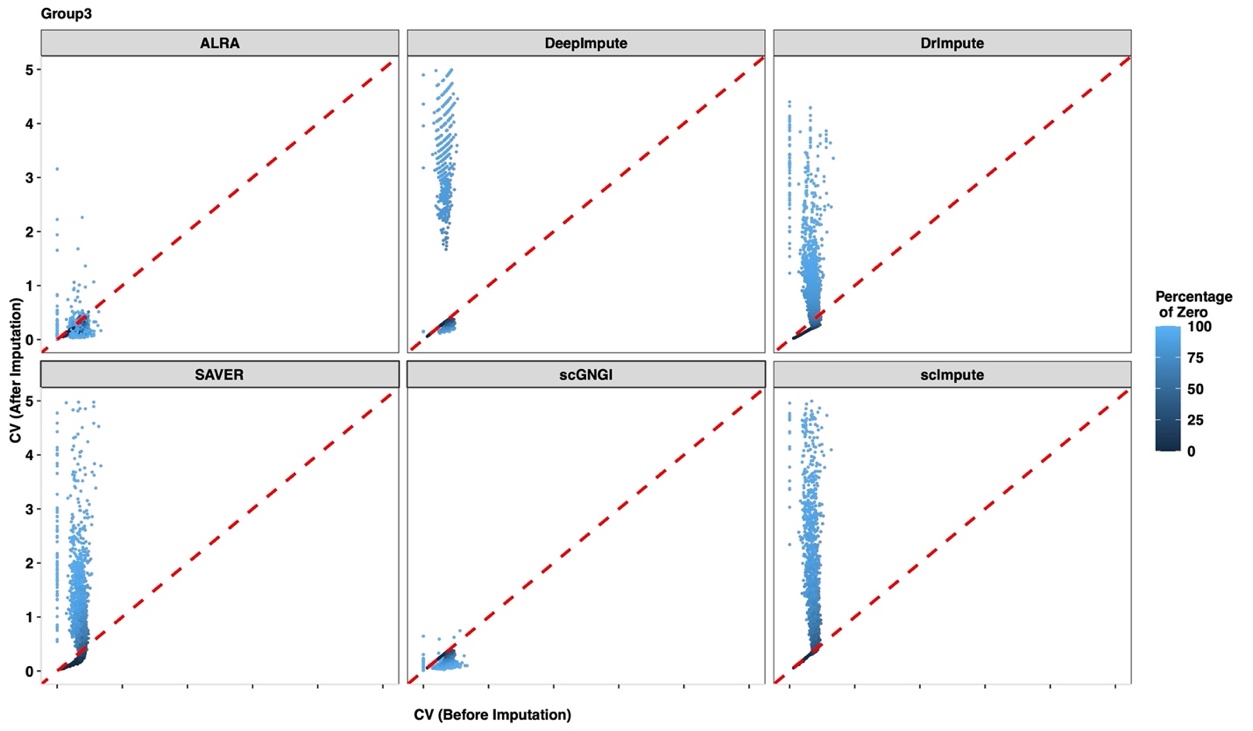


**Supplementary Figure S21.** Gene expression variation between the raw data and the imputed data for Group3 cells in the Simulated Data 1 dataset. The coefficient of variation (CV) is computed for each gene in all Group3 cells after imputation (y-axis) by different methods, and the x-axis represents the CV of non-zero cells before imputation. Each dot represents a gene, and i the mean level of non-zero values is distinguished by its color.


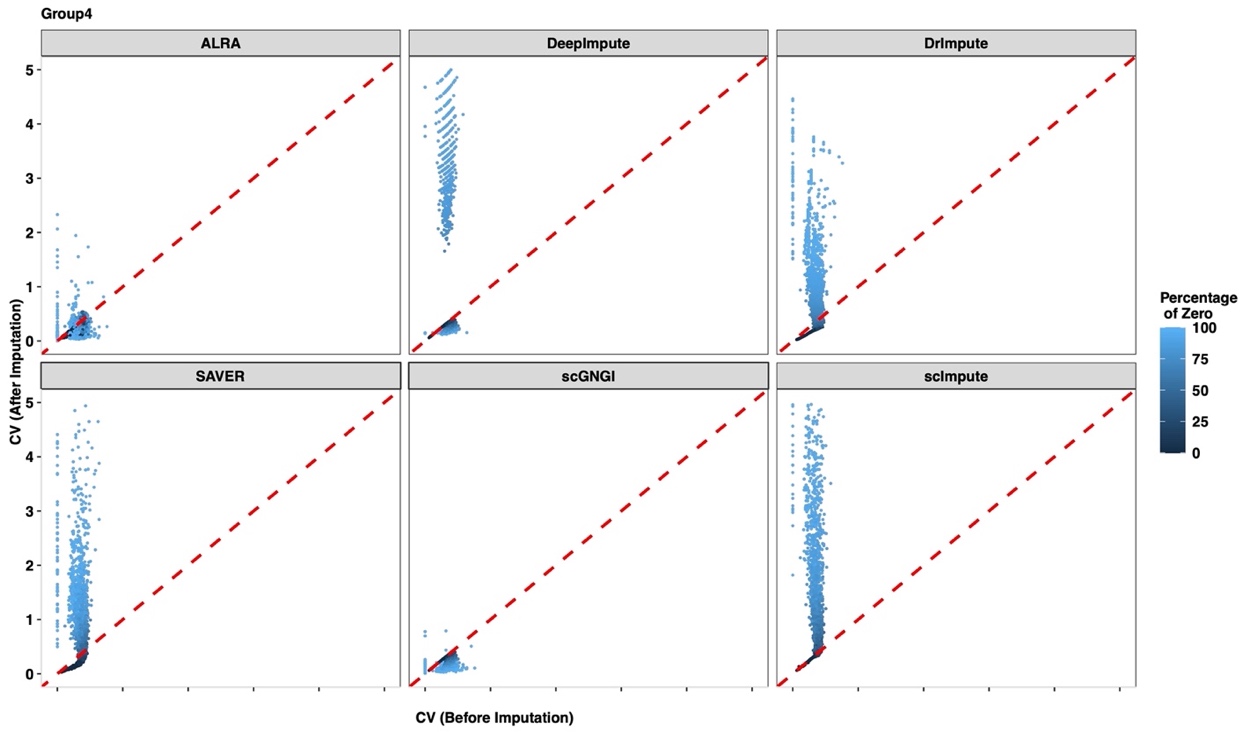


**Supplementary Figure S22.** Gene expression variation between the raw data and the imputed data for Group4 cells in the Simulated Data 1 dataset. The coefficient of variation (CV) is computed for each gene in all Group4 cells after imputation (y-axis) by different methods, and the x-axis represents the CV of non-zero cells before imputation. Each dot represents a gene, and the mean level of non-zero values is distinguished by its color.


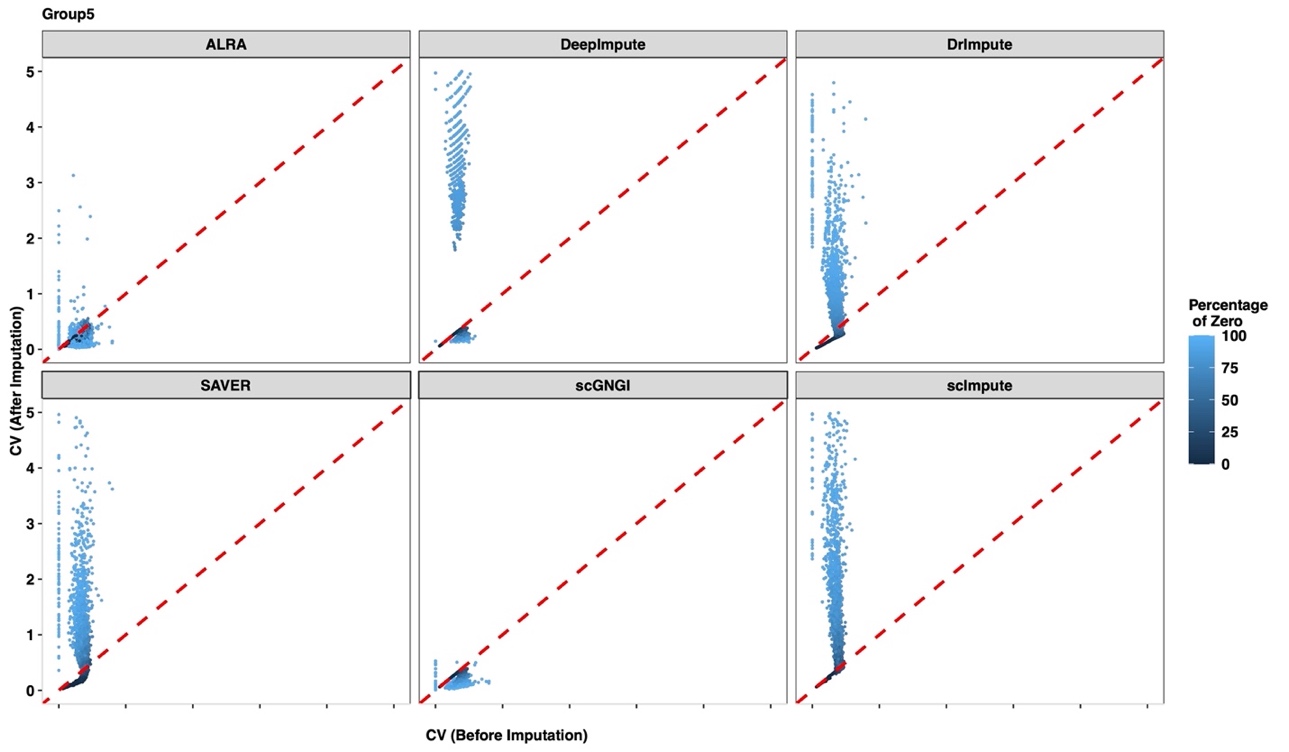


**Supplementary Figure S23.** Gene expression variation between the raw data and the imputed data for Group5 cells in the Simulated Data 1 dataset. The coefficient of variation (CV) is computed for each gene in all Group5 cells after imputation (y-axis) by different methods, and the x-axis represents the CV of non-zero cells before imputation. Each dot represents a gene, and the mean level of non-zero values is distinguished by its color.


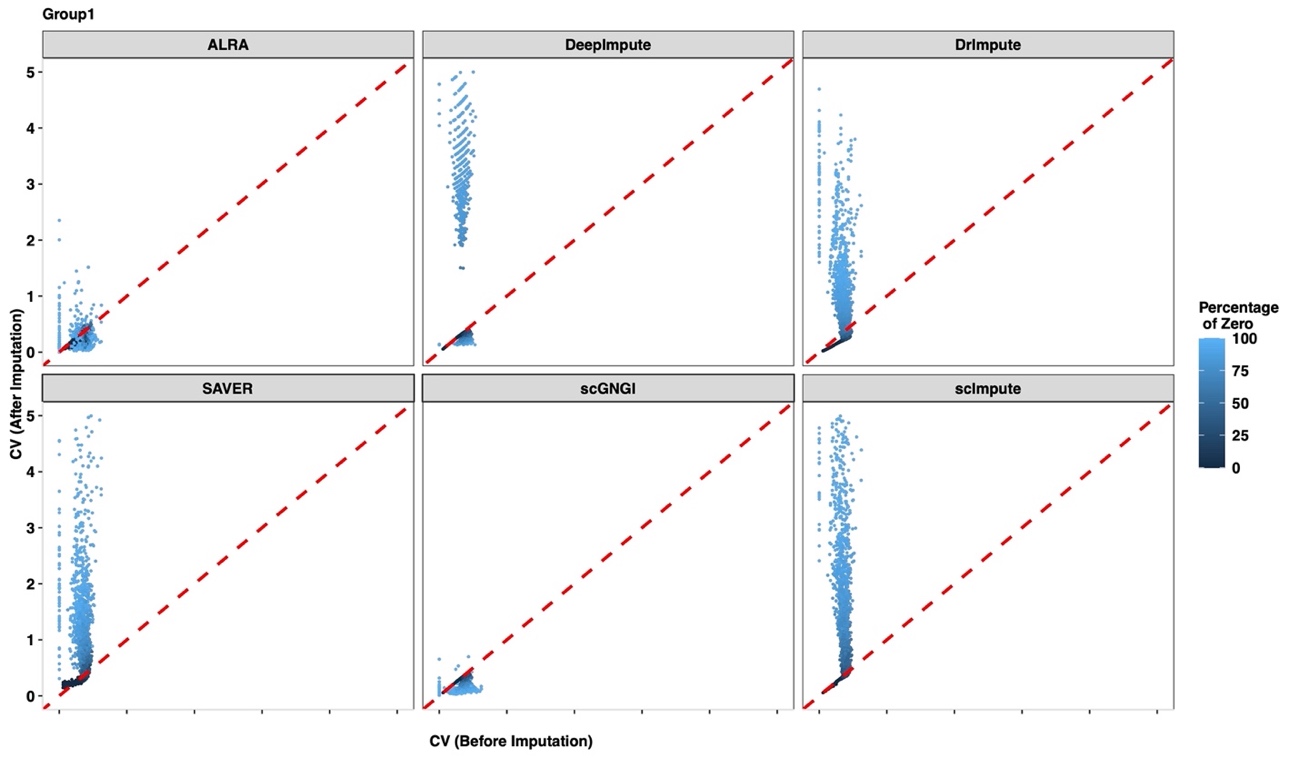


**Supplementary Figure S24.** Gene expression variation between the raw data and the imputed data for Group1 cells in the Simulated Data 2 dataset. The coefficient of variation (CV) is computed for each gene in all Group1 cells after imputation (y-axis) by different methods, and the x-axis represents the CV of non-zero cells before imputation. Each dot represents a gene, and the mean level of non-zero values is distinguished by its color.


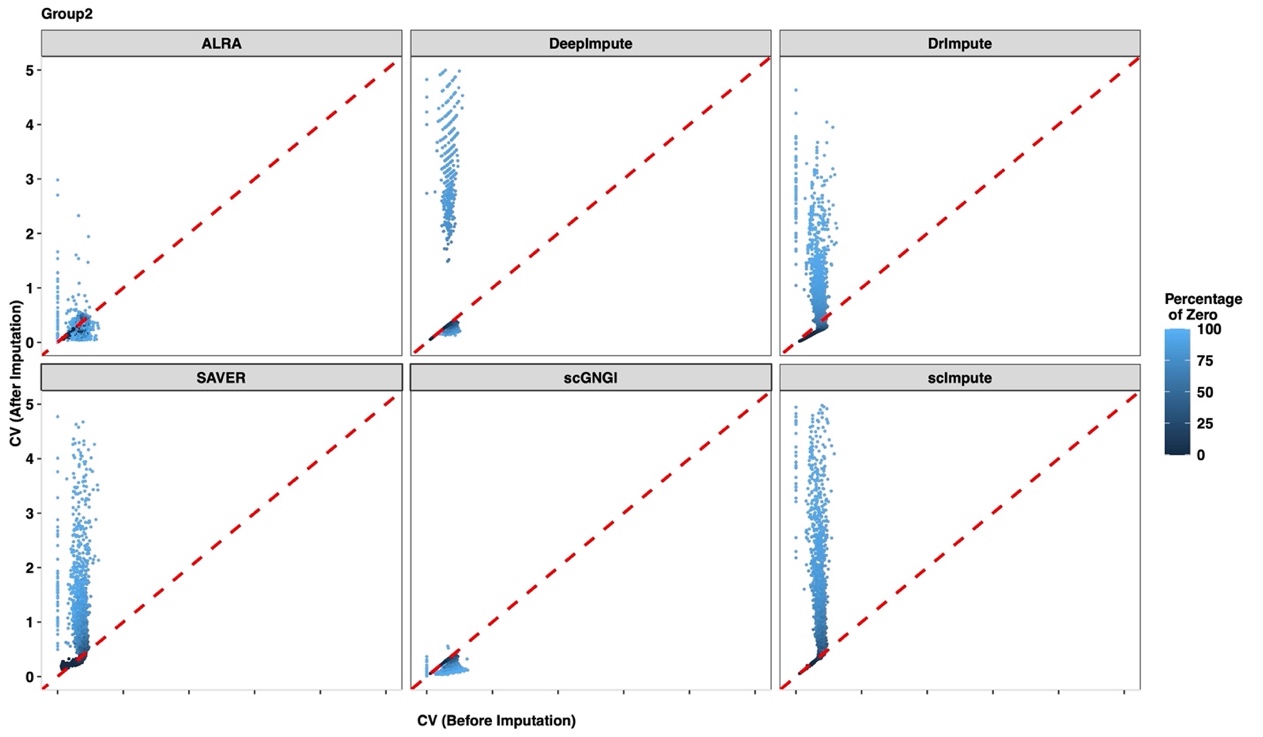


**Supplementary Figure S25.** Gene expression variation between the raw data and the imputed data for Group2 cells in the Simulated Data 2 dataset. The coefficient of variation (CV) is computed for each gene in all Group2 cells after imputation (y-axis) by different methods, and the x-axis represents the CV of non-zero cells before imputation. Each dot represents a gene, and the mean level of non-zero values is distinguished by its color.


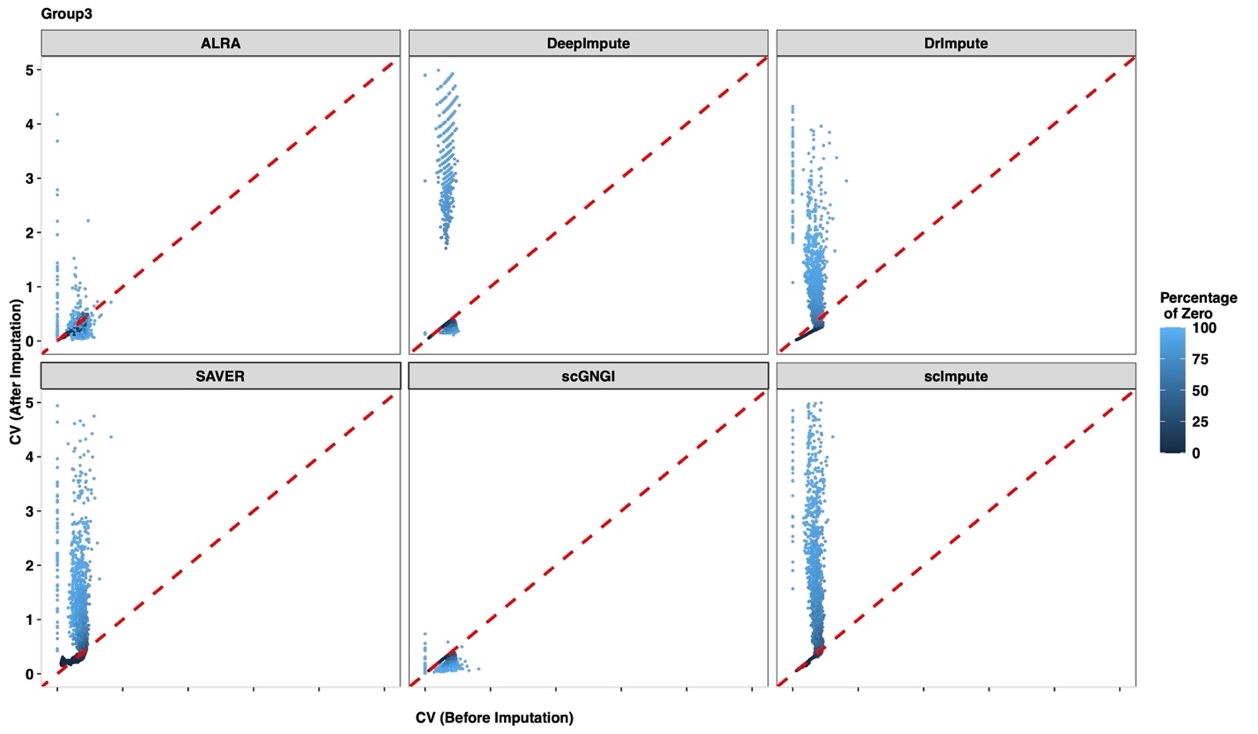


**Supplementary Figure S26.** Gene expression variation between the raw data and the imputed data for Group3 cells in the Simulated Data 2 dataset. The coefficient of variation (CV) is computed for each gene in all Group3 cells after imputation (y-axis) by different methods, and the x-axis represents the CV of non-zero cells before imputation. Each dot represents a gene, and the mean level of non-zero values is distinguished by its color.


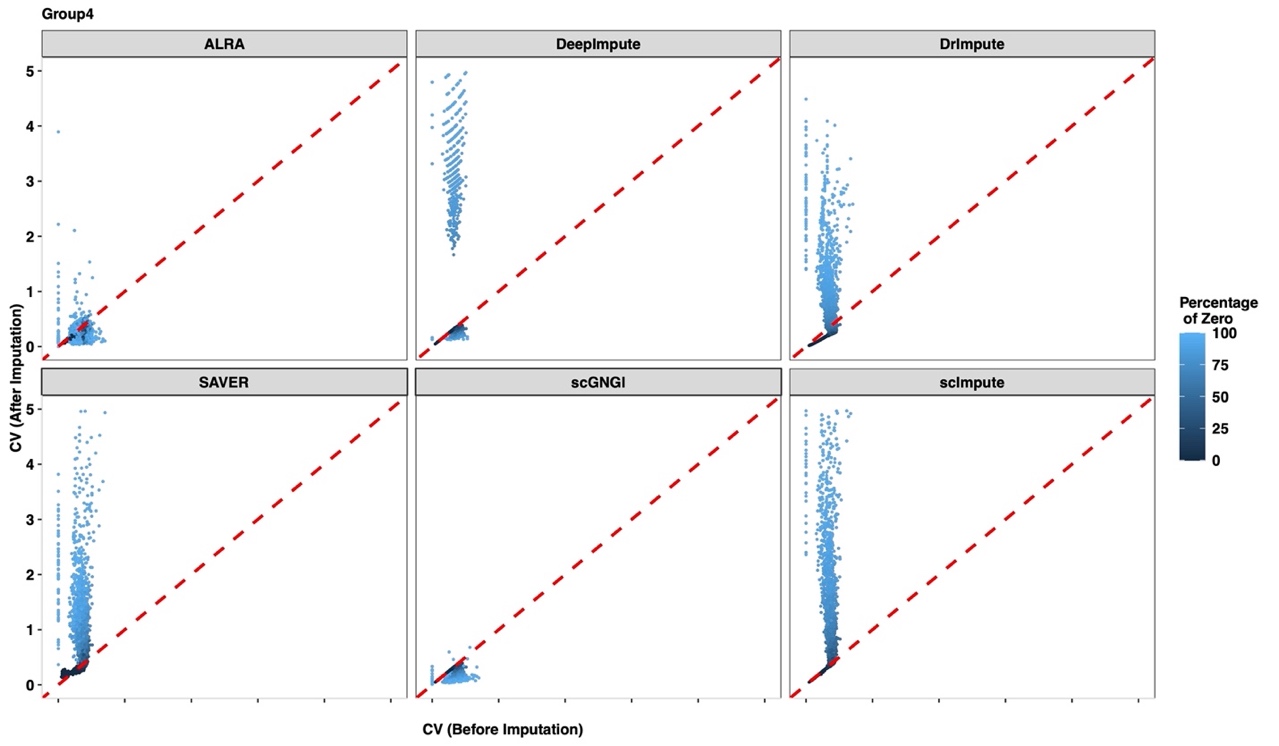


**Supplementary Figure S27.** Gene expression variation between the raw data and the imputed data for Group4 cells in the Simulated Data 2 dataset. The coefficient of variation (CV) is computed for each gene in all Group4 cells after imputation (y-axis) by different methods, and the x-axis represents the CV of non-zero cells before imputation. Each dot represents a gene, and the mean level of non-zero values is distinguished by its color.
